# Prostaglandin E2 breaks down pericyte–endothelial cell interaction via EP1 and EP4-dependent downregulation of pericyte N-cadherin, connexin-43, and R-Ras

**DOI:** 10.1038/s41598-020-68019-w

**Published:** 2020-07-07

**Authors:** Carole Y. Perrot, Jose L. Herrera, Ashley E. Fournier-Goss, Masanobu Komatsu

**Affiliations:** 1 Cancer and Blood Disorders Institute and Institute for Fundamental Biomedical Research, Johns Hopkins All Children’s Hospital, St. Petersburg, FL 33701 USA; 2 Department of Orthopaedic Surgery, Johns Hopkins University School of Medicine, Baltimore, MD 21287 USA

**Keywords:** Cancer microenvironment, Angiogenesis

## Abstract

A close association between pericytes and endothelial cells (ECs) is crucial to the stability and function of capillary blood vessels and microvessels. The loss or dysfunction of pericytes results in significant disruption of these blood vessels as observed in pathological conditions, including cancer, diabetes, stroke, and Alzheimer’s disease. Prostaglandin E2 (PGE2) is a lipid mediator of inflammation, and its tissue concentration is elevated in cancer and neurological disorders. Here, we show that the exposure to PGE2 switches pericytes to a fast-migrating, loosely adhered phenotype that fails to intimately interact with ECs. N-cadherin and connexin-43 in adherens junction and gap junction between pericytes and ECs are downregulated by EP-4 and EP-1-dependent mechanisms, leading to breakdown of the pericyte–EC interaction. Furthermore, R-Ras, a small GTPase important for vascular normalization and vessel stability, is transcriptionally repressed by PGE2 in an EP4-dependent manner. Mouse dermal capillary vessels lose pericyte coverage substantially upon PGE2 injection into the skin. Our results suggest that EP-mediated direct disruption of pericytes by PGE2 is a key process for vascular destabilization. Restoring pericyte–EC interaction using inhibitors of PGE2 signaling may offer a therapeutic strategy in cancer and neurological disorders, in which pericyte dysfunction contributes to the disease progression.

## Introduction

Pericytes are mesenchyme-derived mural cells that surround endothelial cells (ECs) of capillary blood vessels and microvessels. Their physical interaction with ECs is essential for the regulation of EC proliferation and differentiation, vessel contractility and tone, and stability and barrier function of the vessel wall^1–4^. Pericytes are necessary to maintain quiescence of stable endothelium^1,5^, and insufficient pericyte coverage of blood vessels is associated with diverse pathologies, including diabetic retinopathy, Alzheimer’s disease, cardiovascular disorders, and cancer^2,6–10^. Pericytes are frequently detached from blood vessels in tumors destabilizing the tumor vasculature. The vessel destabilization leads to excessive angiogenesis and facilitates penetration of cancer cells into the circulation increasing the risk of metastasis^11–14^. Conversely, increased pericyte coverage of blood vessels is associated with decreased frequency of cancer cell invasion into the circulation^15^. The loss of pericytes in amyloid angiopathy destabilizes cerebral microvasculature and causes plasma leakage and exposure of plasma proteins to neurons, resulting in neurodegeneration^16–21^. The pericyte loss from the cerebral vasculature is thought to be a driving factor for Alzheimer’s disease progression^16–21^.

Pericytes and ECs establish direct contacts within “peg-and-socket”-like membrane structures, and these direct contacts are critical for vessel stability. Ultrastructural analyses of these contacts in intact vessels show that pericytes form cytoplasmic elongations (the “pegs”) that are inserted in the invaginations of the endothelial membrane (the sockets)^22^. Peg-and-socket contacts are highly enriched with N-cadherin-dependent adherens junctions and connexin-43 (Cx43)-dependent gap junctions^23^. The other type of pericyte–EC contact is made of focal adhesion plaques, in which the extracellular matrix connects pericytes with ECs indirectly via integrin-dependent cell adhesion of both cell types^23^. Pericytes and ECs are thought to communicate through these two types of physical contacts. The signal transmission between the two cell types can occur through N-cadherin, which interacts with and activates Trio, a dual Rac1/RhoA guanine nucleotide exchange factor that induces the recruitment of VE-cadherin to EC–EC junctions thereby stabilizing the endothelial barrier^24^. On the other hand, Cx43-mediated gap junctions allow the transfer of ions, second messengers such as cAMP, and other small molecules between pericytes and endothelial cells^1,23,25,26^, and are required for the EC-induced differentiation of mural cell precursors during vessel assembly^27^. Gap junctions play a role in integrating vasopressive signals along the vessel to maintain vascular tone^28^. In contrast, mechanical signals are transmitted through adhesion plaques, such as contractile forces from pericytes to ECs^3,23^. Pericytes and ECs communicate also via paracrine signaling of growth factors (e.g. angiopoietin-1 and PDGF) and their receptors (e.g. Tie-2 and PDGFRβ) as well as juxtacrine signaling (e.g. Jagged1-Notch3)^2,29,30^. Previous studies by our laboratory showed that R-Ras, a small GTPase of the Ras family, plays critical roles in the pericyte–EC interaction^31^. The R-Ras deficiency increases angiogenic response of ECs to VEGF, impairs pericyte association, and exacerbate pathological vessel regeneration^32–34^. On the other hand, R-Ras upregulation in either ECs or pericytes restores the pericyte–EC interaction and stabilizes the integrity of the vessel wall^31^. R-Ras signaling also promotes lumenogenesis of EC via non-canonical Akt signaling that stabilizes microtubules^35^. It is known that R-Ras enhances integrin-mediated cell adhesion to extracellular matrix^36^.

Prostaglandin E2 (PGE_2_) belongs to a family of prostanoid lipids, which are produced by cyclooxygenase (COX)-mediated metabolism of arachidonic acid. Deregulation of COX-2 leads to an excessive and constant release of PGE_2_ in chronic inflammation and often in cancer, facilitating tumor growth and metastasis^37–39^. PGE_2_ is the most abundant prostaglandin found in human cancers, including colon, breast, lung, and pancreatic cancer^40,41^, and high levels of PGE_2_ are associated with poor clinical outcomes^38,42^. Furthermore, high levels of PGE2 are associated with pancreatic fibrosis though the activation of myofibroblast-like stellate cells, a hallmark of chronic pancreatitis and pancreatic cancer^42^. Highly malignant pediatric cancers, such as high-risk neuroblastoma, also exhibit high levels of PGE_2_^43^. Cancer cells as well as stromal cells, such as carcinoma associated fibroblasts, both contribute to the excessive production of PGE2^43–45^.

The activities of PGE_2_ are mediated through four prostanoid receptors, EP1, EP2, EP3 and EP4. The activation of PGE_2_-EP2/4 pathway induces vasodilation, resulting in increased local blood flow and vascular hyperpermeability^46^. In contrast, stimulation of EP_3_ enhances endothelial barrier, resulting in hypopermeability^46^. PGE_2_ also act as a pro-angiogenic molecule, and PGE2 is thought to play an important role in the regulation of tumor angiogenesis and metastasis^47–50^. Several studies have shown that PGE_2_ increases the expression of vascular endothelial growth factor (VEGF) in a variety of cell types, including endothelial cells^51–53^, fibroblasts^54–56^ as well as tumor cells^40,57,58^, thereby promoting angiogenesis. PGE2 also represses *RRAS* gene expression in ECs via CREB3-dependent cAMP signaling, thereby increasing endothelial permeability^59^.

Although PGE2 critically participates in the regulation of angiogenesis and vessel permeability, it remains largely unknown how it affects pericytes and their function. In this report, we studied the effect of PGE2 on pericytes and their interaction with ECs.

## Materials and methods

### Cell culture

Human brain vascular pericytes (HBVP) as well as pericyte complete medium were purchased from Sciencell (Carlsbad, CA). Human umbilical cord vein endothelial cells (HUVECs) and EGM-2 growth medium were obtained from Lonza (Basel, Switzerland). HEK293T cells and HT29 colon cancer cells were from ATCC (Manassas, VA, USA) and cultured respectively in DMEM (high glucose, with L-glutamine) and in McCoy’s 5A modified medium (Gibco, ThermoFisher Scientific, Waltham, MA) both supplemented with 10% FBS. All our experiments involving primary cells were performed using HBVP and HUVECs at passage 3 to 5.

### Antibodies and reagents

For western blot applications, rabbit anti-R-RAS antibody was obtained from AnaSpec (Fremont, CA). Rabbit anti-C-terminal Cx43, anti-phospho-FAK^Y397^, anti-phospho-FAK^Y925^, anti-FAK, anti-phospho-paxillin, anti-paxillin, anti-Src, anti-phospho-MLC2 and anti-MLC2 were purchased from Cell Signaling (Danvers, MA, USA). Mouse anti-N-terminal Cx43 was from EMD Millipore (Burlington, MA, USA). Mouse anti-GAPDH and anti-EP4 were from Santa Cruz Biotechnologies (Santa Cruz, CA). All secondary HRP-conjugated antibodies were obtained from Promega (Madison, WI, USA). For western blot and immunofluorescence staining, mouse anti-N-cadherin was from BD Biosciences (San Jose, CA) and rabbit anti-Cx43 was purchased from Cell Signaling. Rabbit anti-NG2 was from EMD Millipore. For immunofluorescence only, rabbit anti-phospho-CREB was obtained from Cell Signaling. Rabbit anti-phospho-FAK^Y397^ was from ThermoFisher Scientific and mouse anti-Ki67 was from BD Biosciences. Alexa Fluor 647 Phalloidin as well as Alexa Fluor secondary antibodies (488, 555, and 647) were purchased from ThermoFisher Scientific. PGE2 was purchased from Tocris (Bristol, UK). ONO-8713 (EP1 inhibitor), ONO-AE5-599 (EP3 inhibitor) and ONO-AE3-208 (EP4 inhibitor) were provided by Ono Pharmaceuticals (Osaka, Japan). PF-04418948 (EP2 inhibitor), calpain inhibitor XII, PKC inhibitor Gö6976 and cathepsin L inhibitors were from Cayman Chemical (Ann Harbor, MI). Pepstatin A was obtained from Sigma (St Louis, MO, USA).

### Western blot analysis

Western blotting was performed by electrophoresis of cell lysate on Mini-PROTEAN TGX Precast Gels (Bio-Rad, Hercules, CA, USA), followed by electrotransfer to nitrocellulose membrane (Bio-Rad). After blocking unspecific binding, antibody incubations were carried out overnight in blocking buffer (5% BSA or 5% nonfat milk in TBS containing 0.1% Tween-20), and target proteins were detected using Western Lightning Plus-ECL (PerkinElmer, Waltham, MA, USA). Blots were quantified using ImageJ.

### RNA extraction and RT-qPCR

RNA extraction was conducted using Nucleospin RNA plus kit (Macherey–Nagel, Düren, Germany). Between 500 ng and 1 µg of RNA was then subjected to DNase I digestion (ThermoFisher Scientific) followed by reverse transcription using Superscript IV First-Strand Synthesis System (ThermoFisher Scientific). Quantitative polymerase chain reaction (qPCR) was then performed using Power SYBR Green PCR Master Mix (ThermoScientific). Primer sets for *RRAS*, *CDH2*, *CX43*, *EP1*, *EP2*, *EP3*, *EP4*, *PTGES*, and *Cyclophilin A* are shown in Table 1.

**Table 1:** Primers for quantitative RT-PCR.

Target gene	Forward primer	Reverse primer
*RRAS*	5′ TAA CGA CCG GCA GAG TTT CA	5′ ACC AAC ACA ACG GGG AAG TC
*CDH2*	5′ CAC AGC CAC GGC CGT CAT CA	5′ TGG GTC GGT CTG GAT GGC GA
*Cx43*	5′ CAA AAT CGA ATG GGG CAG GC	5′ GCT GGT CCA CAA TGG CTA GT
*EP1*	5′ CAC CTT CTT TGG CGG CTC TC	5′ GAT GCA CGA CAC CAC CAT G
*EP2*	5′ ACC TAC TTC GCT TTC GCC AT	5′ GCT GGT AGA AGT AGG GGT GC
*EP3*	5′ TGG ATC CTT GGG TTT ACC TGC	5′ AGC AGC TGG AGA CAG CAT TT
*EP4*	5′ AGG ACA AGG TGA AAG CAG GTT	5′ AGT GCA AGG CTG GGT CTG TAG
*PTGES*	5′ GTG ACC AGC CAC TCA AAG GA	5′ AGG GGA CAT TTG CAG TTT CCA
*Cyclophilin A*	5′ CAA ATG CTG GAC CCA ACA CA	5′ TGC CAT CCA ACC ACT CAG TCT

### Cell adhesion assay

HBVPs were cultured in 60 mm dishes in the presence of either DMSO or PGE2 (various concentrations) for 72 h, or incubated with conditioned medium from transfected HT29 for 48 h. Cells were washed with 1X PBS, and detached using 0.005% trypsin. Cells were pelleted by centrifugation, resuspended in basal medium and seeded in 24 well-plates (20,000 cells/well). After an incubation for 30 min at 37 °C, unattached cells were removed with PBS and adherent cells were fixed with a 20% methanol/0.5% crystal violet solution for 15 min at room temperature. Crystal violet was extracted from cells using 100 μl of ethanol 100% and absorbance was measured at 595 nm. The assay was repeated three times in quadruplicates for each condition.

### Wound closure assay (scratch assay)

HBVPs were seeded in 6-well plates at the density of 60,000 cells/dish and treated with DMSO or increasing concentrations of PGE2. 72 h post-treatment, a scratch was performed with a 200 μl-pipette tip to create a wound in the confluent cell monolayer. Wound closure was monitored by taking bright field pictures of the cells immediately after scratch and 4 h later using an optical microscope (Nikon Eclipse TS100) and NIS-Elements BR4.40.00 software. The ImageJ software was used to measure wound closure areas. The experiment was repeated three times in two independent wells for each condition. A representative result from three experiments is shown with the analysis of 10 micrographs.

### Transwell migration assay

HBVPs were cultured in 60 mm dishes in the presence of either DMSO or PGE2 (various concentrations) for 72 h. Cells were next washed with PBS, trypsinized and pelleted by centrifugation. After resuspension in basal medium, cells were seeded in 6.5 mm Transwell with 8.0 µm pore inserts (20,000 cells/insert) and incubated for 8 h at 37 °C. The Transwell lower chambers were filled with complete pericyte medium as a chemoattractant. To analyze cell migration, cell culture medium was removed from both upper and lower chambers and inserts were washed twice with 1X PBS. Cells were fixed with formaldehyde 3.7% for 2 min at room temperature, then rinsed twice with 1X PBS. Cell permeabilization was performed using methanol 20% for 20 min at room temperature, followed by staining with a 20%/0.5% crystal violet solution for 15 min at room temperature. Inserts were washed twice with 1X PBS, and non-migrated cells were scraped off from the inner surface of the inserts. Migrated cells were observed using an optical microscope (Nikon Eclipse TS100) and NIS-Elements BR4.40.00 software and quantified by ImageJ software. The experiment was repeated at least three times in two independent Tranwells for each condition. Results are representative of one experiment and display the analysis of 12 micrographs.

### Enzyme-linked immunosorbent assay (ELISA)

To determine the amount of PGE2 produced by colon cancer cells, HT29 were seeded in 12-well plates at the density of 80,000 cells/well for 24 h, then transfected with control or PTGES-targeting siRNA for 48 h before conditioned medium collection. Quantitative determination of PGE2 in culture supernatants was performed using the PGE2 high sensitivity ELISA kit following the manufacturer’s instructions (Enzo Life Sciences, Farmingdale, NY). All conditions were tested in four independent wells, and experiments were repeated at least twice.

### Live cell Fluo4-calcium assay

For confocal imaging of intracellular calcium (Ca^2+^), HBVPs were seeded on glass-bottom dishes (MatTek, Ashland, MA, USA). At 70% confluence, primary cells were treated with either DMSO or PGE2 (10 nm or 100 nm) for 24 and 72 h. Following treatment, cells were washed with Krebs–Ringer-HEPES buffer and incubated with Fluo-4 acetoxymethyl (AM) (5 mM; Invitrogen) for 40 min. After successive washes and incubation with KRH for 30 min, the level of intracellular Ca^2+^ in pericytes was assessed by basal Fluo-4 fluorescence imaging using a laser confocal microscope (Nikon Eclipse Ti2, Nikon Instruments, Melville, NY). Processing and analysis of the acquired images were carried out using Nikon NIS-Elements AR Analysis 4.40 software. Resting cytosolic Ca^2+^ levels were measured using the ROI measurement tool, considering each pericyte a single ROI in selected multi-regions of interest (multi-ROIs). Approximately 600–700 cells were scored for each experimental condition.

### Lentivirus transduction

For reporter assays, HBVP were stably transduced with pLenti6-R4R2-*RRAS*-1907/ + 1-Fluc lentivirus (MOI 0.5; hexadimethrine bromide 10 µg/ml). For R-Ras rescue experiments, cells were transduced with a lentiviral expression vector carrying cDNA for a constitutively active form of R-Ras (R-Ras38V), or insertless control (mock) as described before^32^. The day after transduction, media was replaced with fresh media, and the cells were cultured for 3 days before the use for experiments.

### RNA interference

For gene silencing, cells were transfected at approximately 60% confluency in complete medium with 30 nM siRNA targeting EP1*, EP4** or PTGES* (*Dharmacon, Lafayette, CO; **ThermoFisher;) using Lipofectamine RNAiMAX Reagent (ThermoFisher Scientific) or Dharmafect (Dharmacon). Cells were incubated with fresh cell culture medium 24 h later and cultured for 48 additional hours before analyses were performed.

### Luciferase assay

As the efficiency of transfection in primary cells is poor, HBVPs were stably transduced with the *RRAS*-1907/+ 1-Fluc lentivirus and then seeded in 96-well plates at a density of 6,000 cells/well. Luciferase activity was measured 24 h after PGE2 treatment using the Steady-Glo luciferase kit from Promega. The measurement of cell viability using the CellTiter-Fluor kit (Promega) served as an internal control. Transient transfections were performed in 293 T cells to assess *RRAS* promoter activity. Cells were first seeded in 96 well-plates at the density of 20,000 cells/well. The next day, cells were co-transfected with either a wild-type *RRAS*-1907/+ 1-Fluc promoter construct or a mutated construct without cyclic AMP-responsive elements as well as a Renilla luciferase reporter plasmid to monitor transfection efficiency. Lipofectamine LTX with Plus Reagent (ThermoFisher Scientific) was used as a plasmid transfection reagent according to the manufacturer’s instructions. Luciferase activity was measured 24 h after PGE2 treatment using the Dual-Glo luciferase kit from Promega.

### Pericyte–EC coculture models

PGE2- or conditioned medium-treated HBVPs and untreated HUVECs were cultured for 48 h, then incubated for 30 min at 37 °C with 8 μM CellTracker Green CMFDA and CellTracker Red CMPTX dyes (ThermoFisher Scientific), respectively. Then cells were washed with 1X PBS, and incubated with fresh complete cell culture media for additional 24 h (pericytes were maintained in the presence of exogenously added PGE2 in the corresponding experiment). In the 2-D coculture model, HBVPs and HUVECs were seeded in 8-well Lab-Tek chamber slides at the density of 6,600 and 20,000 cells/well (ratio HUVECs-pericytes 3:1), respectively. Cell monolayers were processed for N-cadherin or Cx43 immunofluorescence staining 24 h later. In the 3-D coculture model, cells were seeded at the same density as above in 8-well Lab-Tek chamber slides coated with phenol-red free, non-growth factor reduced Matrigel. Cells were fixed 16 to 18 h later using 4% paraformaldehyde (PFA), mounted with coverslips and analyzed using a fluorescence microscope (Nikon Eclipse 90i). Images were captured using NIS-Elements BR3.2 software and analyzed using ImageJ software. For the 3-D coculture analysis, we used Angiotool^60^ for automated measurement of the branching index and average vessel length. The number of vessels was determined using ImageJ.

### Immunofluorescence

Cell monolayers were fixed with 4% (PFA) for 10 min, then permeabilized using PBS solution containing 5% FBS and 0.1% Triton X-100 for one hour at room temperature. Cells were incubated with mouse anti-N-cadherin (1/200 dilution), rabbit anti-connexin antibody (1/200 dilution), rabbit anti-phospho-CREB1 (1/200 dilution) or rabbit anti-phospho-FAK^Y397^ (1/100 dilution) overnight at 4 °C, followed by incubation with anti-mouse or anti-rabbit Alexa Fluor 488 secondary antibody (1/1,000) along with, when applicable, Alexa Fluor 647 Phalloidin for actin staining for 2 h at room temperature. Cells were finally incubated with DAPI for 5 min and slides were then mounted with coverslips. Signals were visualized using a fluorescence microscope (Nikon Eclipse 90i). Images were captured using NIS-Elements BR3.2 software and analyzed using ImageJ software.

### In vivo pericyte/EC association study

All animal experiments were approved by the Institutional Animal Care and Use Committee (IACUC) of Johns Hopkins University. All methods were performed in accordance with the relevant guidelines and regulations. Eight-week-old C56BL/6 mice were injected intradermally with either vehicle (30% DMSO in PBS) or PGE2 (200 ng) on Day 0 and Day 1 in the same area of dorsal skin. On Day 2 (48 h from the first PGE2 injection), mice were sacrificed and skin samples were collected and cryopreserved in OCT compound for preparation of tissue sections. Tissue sections were fixed with 4% PFA for 15 min at room temperature followed by incubation in blocking buffer (10% goat serum, 3% BSA in PBS) for one hour at room temperature, then immunostained for CD31 (1/200) and NG2 (1/200), overnight at 4 °C. Next, tissue sections were rinsed and incubated with anti-rabbit Alexa Fluor 555 and anti-rat Alexa Fluor 647 secondary antibodies (1/500) for two hours at room temperature. Fluorescence images were captured with a Nikon Eclipse Ti2 confocal microscope and analyzed using NIS-Elements AR 5.11.00 software. Pericyte coverage was determined as the percentage of NG2^+^CD31^+^ double-positive area in the total CD31^+^ area of each vessel. Quantitative analysis was conducted in a total of > 68 micrographs of different areas in five skin samples.

### Statistical analyses

Statistics were performed using GraphPad Prism 8. The two-tailed Student’s t test was used to compare two conditions. For multiple comparisons, we used either the one-way, two-way or three-way ANOVA with Dunnett’s or Tukey’s multiple comparison test as appropriate for each comparison. Error bars represent standard error of mean (S.E.M.).

## Results

### Pericytes exposed to PGE2 display pronounced morphological and phenotypic changes

**Figure 1:**
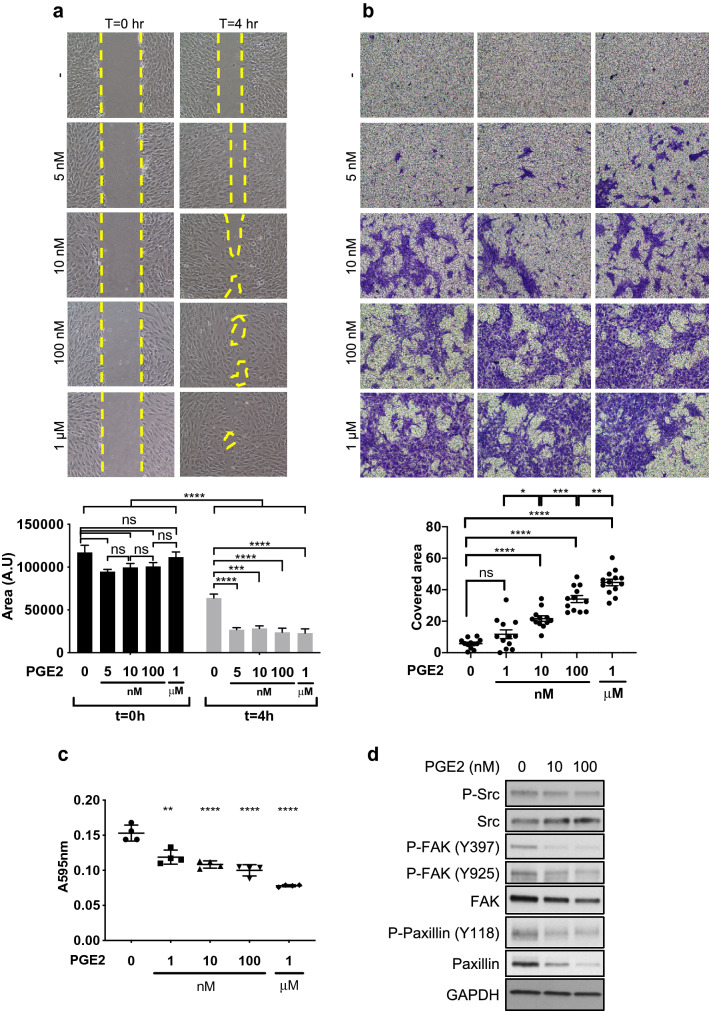
PGE2 alters proliferation, migration, survival and adhesion of pericytes. (**a**) Human brain microvascular pericytes (HBVPs) were cultured for 72 h with increasing concentrations of PGE2 and a wound closure assay was performed to assess their migration activity. The areas of uncovered surface were determined in 9 micrographs from 3 different dishes using ImageJ software. Representative data from three independent experiments performed in triplicates are shown. (**b**) PGE2-treated HBVPs were seeded in Transwell inserts for migration assay. Eight hours later, the migrating cells were fixed and stained. The stained area was measured in 12 micrographs from 4 Transwells for each condition. A representative result of two independent experiments performed in quadruplicates is shown. (**c**) HBVPs were incubated with various concentrations of PGE2 for 72 h, detached from the cell culture dish by 0.005% trypsin and seeded in 24-well plates for 30 to 45 min in basal medium, at 37 ºC. Unattached cells were removed by PBS wash, and cells were fixed and stained with methanol/crystal violet solution. Crystal violet was subsequently extracted from attached cells to measure the absorbance at 595 nm. The experiment is representative of three experiments performed in quadruplicates. (**d**) HBVPs were treated with either control DMSO, PGE2 10 nM or PGE2 100 nM for 72 h before protein extraction. Western blot analysis was performed to assess the expression of proteins involved in cell adhesion: Src/phospho-Src, FAK/phospho-FAK and paxillin/phospho-paxillin. GAPDH was used as an internal control. One-way ANOVA, *p < 0.05; **p < 0.01; ***p < 0.001; ****p < 0.0001; ns, not significant.

PGE2 has been implicated in blood vessel instability as it promotes angiogenesis and increases vascular permeability. We investigated the effects of PGE2 on pericytes. Human brain microvascular pericytes (HBVP) were cultured in increasing concentrations of PGE2 for 72 h in order to recapitulate pathological conditions, such as tumor microenvironment, in which pericytes are exposed to high levels of environmental PGE2 for an extended time. In this study, we observed that the PGE2 exposure results in marked changes of the pericyte morphology. They displayed numerous, thin dendrite-like structures with reduced contact area between the cells (Fig. S1a). This morphological change was associated with greatly increased motility, as shown by wound closure and Transwell migration assays (Fig. 1a, b). The overly motile activity of PGE2-exposed pericytes may explain the pericyte detachment from vessels observed in pathological conditions with high PGE2 levels. The contractility of pericytes does not appear to be significantly altered by PGE2 as gel contraction assay did not show any difference between untreated and treated pericytes (Fig. S1b). However, the phosphorylation of myosin-light chain 2, which indicates actomyosin contraction, was reduced, suggesting that pericytes may be less contractile following the exposure to PGE2 (Fig. S1c). This observation is consistent with previous reports demonstrating relaxant properties of PGE2 in vascular cells and other cell types^61–64^.

Next, we assessed pericyte adhesion to the culture plate after 72-h exposure to PGE2. We found that pericyte adhesion was reduced by PGE2 in a dose-dependent manner (Fig. 1c). This result was consistent with the reduced expression and/or tyrosine phosphorylation of various proteins involved in integrin-mediated cell adhesion, including phospho-Src, FAK, and paxillin (Figs. 1d, S1d, S2a). Similar responses of pericytes were observed following exposure to forskolin, an adenylate cyclase/cAMP pathway activator that mimics the effects of PGE2 (Fig. S2b). Phosphorylation of a cAMP-responsive element binding protein, CREB1, was observed in pericytes after PGE2 exposure, confirming the upregulation of cAMP signaling (Fig. S2c). These results suggest that cAMP signaling downstream of PGE2 prevents pericytes from tightly adhering to the basement membrane and stably interacting with ECs.

### PGE2 disrupts pericyte–EC interaction

**Figure 2:**
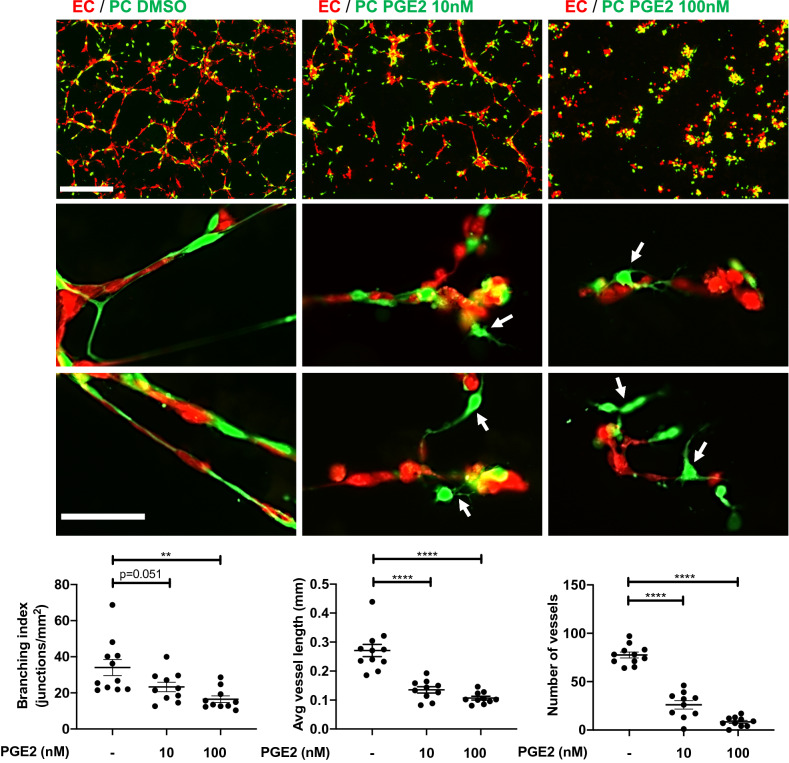
PGE2 impairs pericyte–EC interaction in a 3D-coculture model. HBVPs were treated with PGE2 (10 or 100 nM) or DMSO for 72 h, stained with CellTracker Green and subsequently cocultured with CellTracker Red-stained HUVECs on Matrigel. The association of pericytes with endothelial cells was observed 16–18 h later by fluorescence microscopy. The upper row shows pictures captured with a 4 × objective (scale bar: 500 μm). Middle and lower rows are high magnification observations of pericyte–endothelial cell interactions. Untreated pericytes are closely associated with endothelial cells. White arrows indicate the loss of contact between PGE2-treated pericytes and endothelial cells. Scale bar: 100 μm. The branching index (number of junctions / mm^2^), average vessel length and number of vessels were measured using Angiotool and ImageJ. One-way ANOVA test, **p < 0.01; ****p < 0.0001.

The direct physical contact of pericytes and ECs is essential for communication between the two cell types, and required for the vessel formation, maintenance, and stabilization. We investigated how PGE2 exposure affects pericyte interaction with ECs in 3-D coculture. Pericytes were first exposed to PGE2 (10 or 100 nM) or control DMSO for 72 h, then cocultured with HUVECs on Matrigel without the addition of exogenous PGE2. Eighteen hours later, ECs cocultured with control pericytes formed well-organized vessel-like network of endothelial cords ensheathed by pericytes (Fig. 2). In striking contrast, ECs failed to form a network of endothelial cords and their interaction with pericytes was disrupted when pericytes were pre-exposed to PGE2 (Fig. 2). It is known that ECs alone can form network without pericytes in 3-D culture^65^ (Fig. S2d). Therefore, our results suggest that PGE2-exposed pericytes compromise the ability of ECs to form nascent vessel structure during angiogenesis.

**Figure 3:**
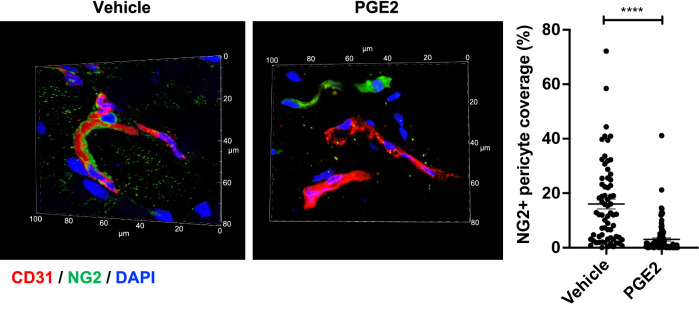
Dermal capillary vessels lose pericyte coverage upon local PGE2 injection. Mice were injected with PGE2 (200 ng) or vehicle (DMSO) into dorsal skin on Day 0 and Day 1, then skin samples were collected and tissue sections were immunostained for NG2 (green), CD31 (red) and DAPI (blue). A representative micrograph of microvessels is shown for each condition. The pictures were taken using a 60 × objective lens. The depth of confocal scanning was 4.35 μm; therefore, winding microvessels are seen in fragments in these pictures. The right picture panel shows NG2^+^ pericytes that are dissociated from the CD31^+^ endothelium in the PGE2-treated skin. Blood vessel coverage by NG2^+^ pericytes is presented in a graph with an arbitrary unit. The analysis was conducted in total of > 68 micrographs of different areas in 5 skin samples for each condition. Each dot represents one micrograph. Student *t*-test, ****p < 0.0001.

Next, we examined pericyte–EC interaction in intact mouse dermal microvessels after PGE2 exposure. Mice received an injection of PGE2 (200 ng) or vehicle intradermally in the dorsal skin, which was repeated once in the same dorsal area 24 h later. The mouse skin at the injection site was examined 48 h post-first injection by immunostaining for EC marker CD31 and pericyte marker NG2. We found that the dermal capillaries in the PGE2-injected skin display substantially reduced association of NG2^+^ pericytes (Fig. 3) consistent with the disruption of pericyte–EC interaction as we observed in vitro. The observed loss of pericyte–EC association is also consistent with the PGE2-induced blood leakage from dermal capillary vessels that we reported previously^59^.

### Inhibition of EP receptors blocks the effects of PGE2 on pericytes.

**Figure 4:**
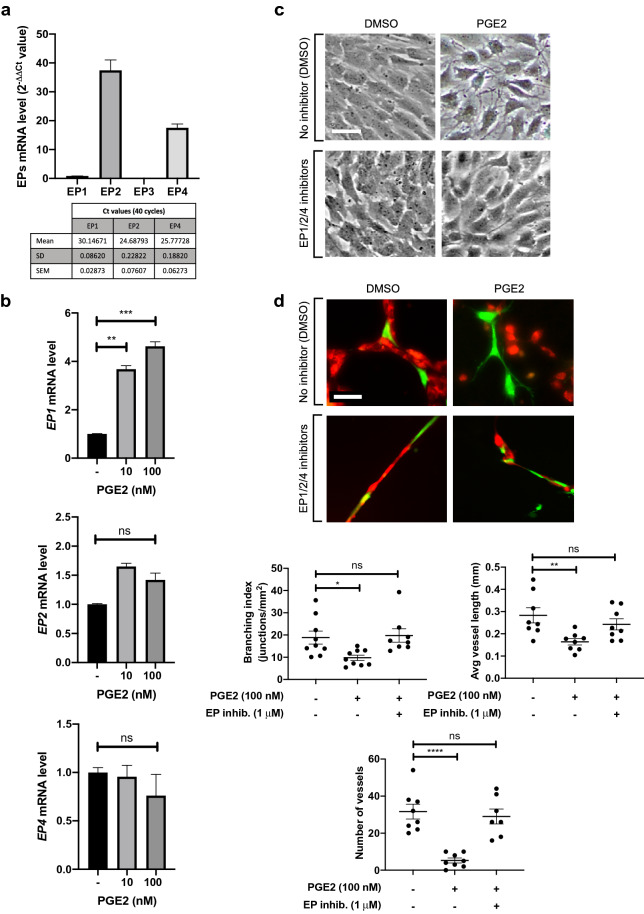
Inhibition of EP1, EP2 and EP4 restores pericyte–EC interaction. (**a**) RT-qPCR analysis of EP receptor expression profile in HBVPs. The different primer sets were validated for an efficiency of 2 ± 5%. EP3 expression was undetectable. (**b**) The effect of PGE2 on EP1, EP2 and EP4 expression in HBVPs was assessed by RT-qPCR 72 h post-treatment. The mRNA expression level of each EP was normalized to that of cyclophilin A using the delta-delta Ct method. Fold change relative to the control is shown. (**c**) HBVPs were treated with a cocktail of EP1 (ONO-8713), EP2 (PF-04418948) and EP4 (ONO-AE3-208) inhibitors (1 μM each) in DMSO or DMSO vehicle alone, then 5 h later with 100 nM PGE2 in DMSO or DMSO alone. Cell morphology was observed 72 h later. Scale bar 50 μm. (**d**) HBVPs (green) subjected to the same treatments as described in c were co-cultured on Matrigel with HUVECs (red) for 18 h. Scale bar 50 μm. The branching index (number of junctions/mm^2^), average vessel length and number of vessels were measured using Angiotool and ImageJ. One-way ANOVA test, *p < 0.05; **p < 0.01; ***p < 0.001; ****p < 0.001; ns, not significant.

PGE2 can activate four prostanoid receptors, EP1-4. EP1, which is coupled to phospholipase C (PLC)/inositol trisphosphate (IP3) signaling, leads to the mobilization of Ca^2+^ from the endoplasmic reticulum, resulting in an increase in intracellular Ca^2+^. EP2 and EP4 activate adenylate cyclase to increase the production of cAMP whereas EP3 has an opposite effect. We examined the expression level of each receptor in unstimulated pericytes by RT-qPCR (Fig. 4a). We found that EP2 and EP4 are the most abundantly expressed as shown by low Ct values, and their expression is unchanged after exposure to PGE2 (Fig. 4b). In comparison, EP1 is expressed at a much lower level in the absence of PGE2 stimulation, but its expression is increased approximately 4 times in PGE2-treated cells (Fig. 4b). Interestingly, the expression of EP3 was undetectable in both PGE2-treated and untreated pericytes. We treated pericytes with specific inhibitors targeting EP receptors. We found that a cocktail of EP1/2/4 specific inhibitors^66–71^ prior to PGE2 treatment allows pericytes to resume the morphology of untreated control cells (Fig. 4c) and intimately associate with ECs in 3-D coculture, thus restoring their capacity to form endothelial cords (Fig. 4d). These results demonstrate that the effects of PGE2 on pericytes are mediated via EP1, EP2 or EP4, but not EP3.

### PGE2 transcriptionally represses R-Ras expression in pericytes in an EP4/cAMP dependent pathway

**Figure 5:**
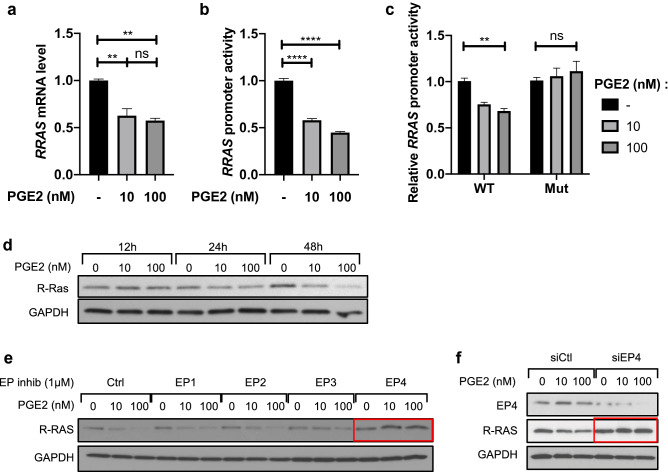
PGE2 transcriptionally represses *RRAS* expression in an EP4-dependent manner. (**a**) HBVPs were treated with PGE2 (10 or 100 nM) or control DMSO for 48 h, and *RRAS* expression was analyzed by quantitative RT-PCR at 48 h post-treatment. The mRNA extracts were pooled from three different cell culture dishes, and the RT-PCR analyses were repeated in two independent experiments and in triplicates. The *RRAS* mRNA level was normalized to that of cyclophilin A using the delta-delta Ct method. Fold change in *RRAS* expression is shown relative to the control. (**b**) In parallel, HBVPs were stably transduced with lentiviral particles delivering a -1907/ + 1 *RRAS* promoter-reporter construct. Luciferase assay was conducted to determine the promoter activity 48-h post-treatment of HBVPs with the same compounds. Luciferase assay results are shown as means ± SEM of three independent experiments performed in tri- or quadruplicates. (**c**) The specificity of *RRAS* promoter activity in response to PGE2 was assessed by transient transfection of 293 T cells with a wild-type (WT) *RRAS* promoter-reporter construct or a mutated construct (Mut) lacking cyclic AMP-responsive elements (CRE). Renilla luciferase activity was used as an internal control. The results are representative of two transfections performed in triplicates. One-way ANOVA test, **p < 0.01; ****p < 0.0001; ns: not significant. (**d**) Time-course western blot analysis of R-Ras expression in HBVPs treated with PGE2 for 12, 24 or 48 h. (**e**) HBVPs were incubated for 5 h with inhibitors of different EP receptors (EP1: ONO-8713; EP2: PF-04418948; EP3: ONO-AE5-599; EP4: ONO-AE3-208) prior to PGE2 treatment. 72 h later, R-Ras protein expression was analyzed by western blot. (**f**) EP4 expression was silenced in HBVPs using siRNA. Cells were treated with PGE2 24 h after transfection, and protein extracts were collected 48 h later for western blot. GAPDH was used as a loading control.

The Ras small GTPase R-Ras plays an important role in blood vessel stabilization, and R-Ras deficiency results in increased vascular permeability due to disrupted EC barrier function and impaired pericyte coverage of blood vessels^72^. We previously reported that the cAMP signaling stimulation by PGE2 transcriptionally downregulates R-Ras and disrupts endothelial barrier function^59^. To examine whether a similar effect is observed in pericytes, we first treated cultured pericytes with PGE2 (10 or 100 nM) for 48 h and analyzed *RRAS* mRNA expression (Fig. 5a) and *RRAS* promoter activity (Fig. 5b). We observed that the *RRAS* mRNA level and promoter activity decreased following PGE2 treatment. The specificity of *RRAS* promoter activity was confirmed in 293 T cells by using a promoter mutant construct that lacks cyclic AMP-responsive elements (CRE) (Fig. 5c). The downregulation of R-Ras expression in response to PGE2 was confirmed at the protein level by Western blot analysis. Pericytes were treated with PGE2 for 12, 24 or 48 h. R-Ras protein expression was not affected 12 h or 24 h post-treatment. However, 48 h after exposure to PGE2, R-Ras expression decreased in a dose-dependent manner (Figs. 5d, S3a).

To identify the EP receptor responsible for the PGE2-mediated *RRAS* repression, we inhibited the different EP receptors using specific inhibitors^66–71,73,74^ prior to PGE2 treatment and observed the expression of R-Ras protein 72 h later (Figs. 5e, S3b). This study demonstrated that EP4 is responsible for the R-Ras downregulation by PGE2. This finding was confirmed by EP4 knockdown using siRNA (Figs. 5f, S3c). In contrast, the inhibition of EP2, which shares downstream signaling with EP4, didn’t restore R-Ras expression. This is likely because the affinity of PGE2 to EP4 is about 22-fold higher than to EP2^75^.

### PGE2 disrupts adherens and GAP junctions between pericytes and ECs

**Figure 6:**
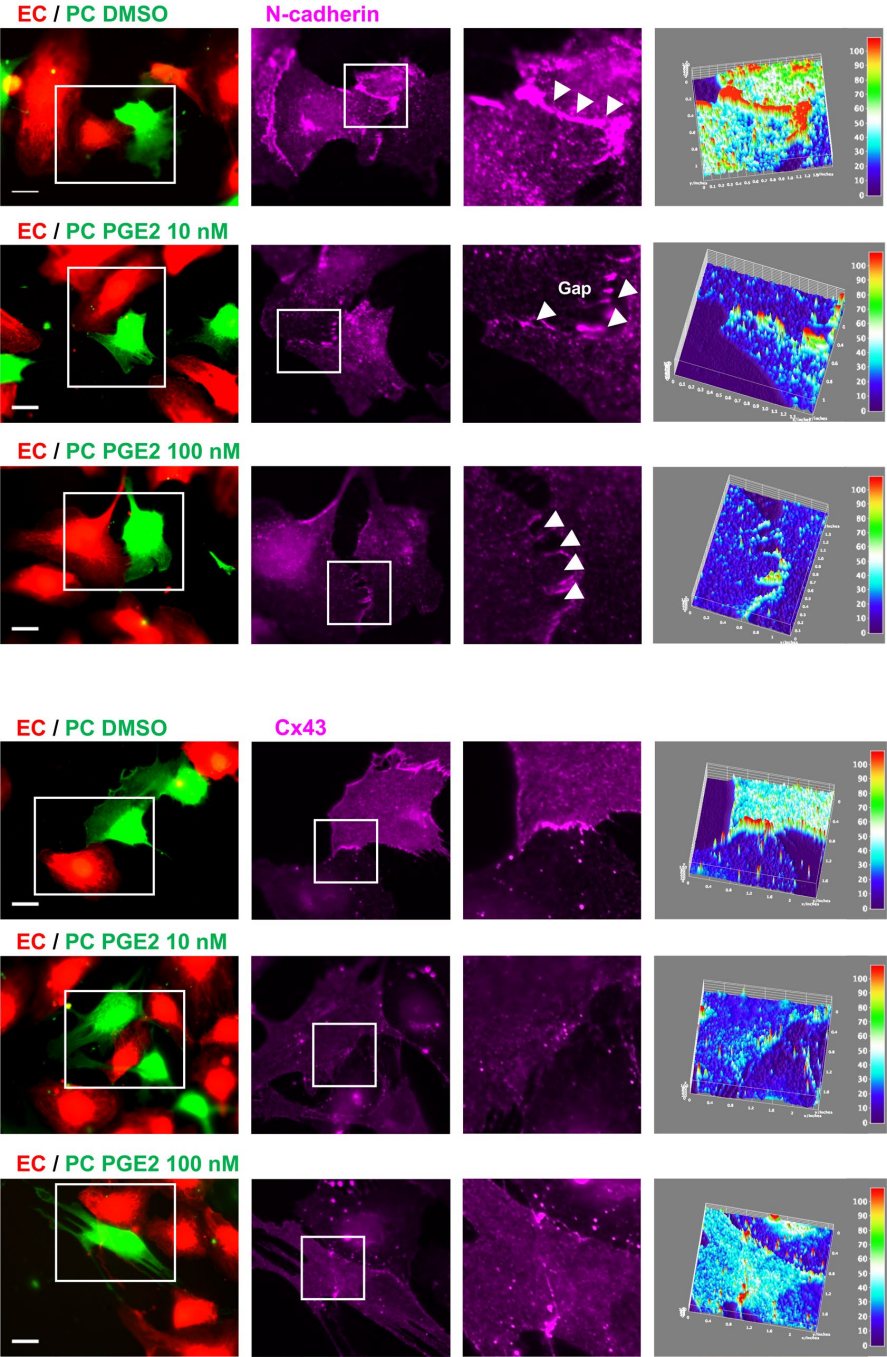
PGE2 impairs N-cadherin and Cx43-mediated pericyte–EC interaction. HBVPs (green) were treated with PGE2 for 72 h prior to coculture with HUVECs (red) on tissue culture plates. Cells were stained for N-cadherin (top panels) and Cx43 (bottom panels) 18 h later. Immunofluorescence intensities of these junctional proteins are shown in surface plots with a color-coded scale created by Image J (right panel, red for the strongest and dark blue for the weakest fluorescence signals). At least 10 different areas of the coculture were analyzed for each condition. A representative area is shown for each. Scale bar 50 μm.

**Figure 7:**
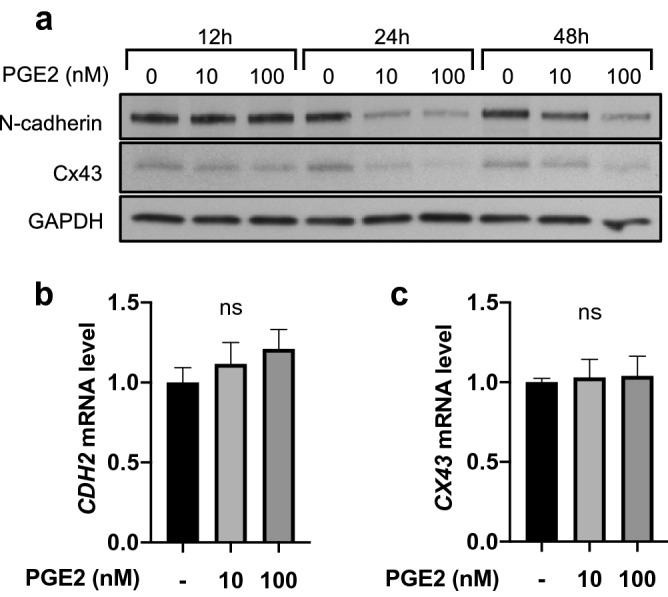
PGE2 downregulates N-Cadherin and Cx43 expression at the protein level. (**a**) Time-course western blot analysis of N-cadherin and Cx43 expression in HBVPs treated with PGE2 for 12, 24, or 48 h. GAPDH was used as a loading control. (**b**, **c**) HBVPs were treated with PGE2 or control DMSO. N-cadherin (**b**) and Cx43 (**c**) expression was then analyzed by quantitative RT-PCR at 48 h post-treatment. The mRNA extracts were pooled from three different cell culture dishes, and the RT-PCR was performed in triplicates, and repeated in two independent experiments. The mRNA expression levels were normalized to cyclophilin A. Fold change is shown relative to the control. One-way ANOVA test, ns, not significant.

Pericytes are embedded within the endothelial basement membrane and directly interact with ECs at peg-socket-like structures containing tight-, gap-, and adherens junctions between the two cell types. To examine how intercellular junctions are affected by pericyte exposure to PGE2, we observed pericyte–EC interaction in a 2-D coculture. In control culture, pericytes and ECs established robust and continuous contacts, as shown by N-cadherin and Cx43 staining (Fig. 6). In contrast, these contacts were disrupted, displaying numerous intercellular gaps when pericytes are pre-exposed to PGE2 before coculture (Fig. 6). The defective pericyte–EC interaction was accompanied by a marked decrease in both N-cadherin and Cx43 accumulation at the cell–cell junctions (Fig. 6). The effect of PGE2 on N-cadherin and Cx43 in pericytes was confirmed by Western blot of pericyte lysate. We observed that both proteins are downregulated in a dose-dependent manner in response to PGE2 as early as 24 h post-treatment (Figs. 7a, S4). Subsequent RT-qPCR analysis revealed that PGE2 does not control N-cadherin and Cx43 expression at the transcriptional level, as shown by the unchanged mRNA level of *CDH2* and *Cx43* following exposure to PGE2 (Fig. 7b,c). PGE2 thus downregulates N-cadherin and Cx43 expression post-transcriptionally in pericytes.

### Cx43 is downregulated upon PGE2/EP1/Ca^2+^/calpain pathway activation

**Figure 8:**
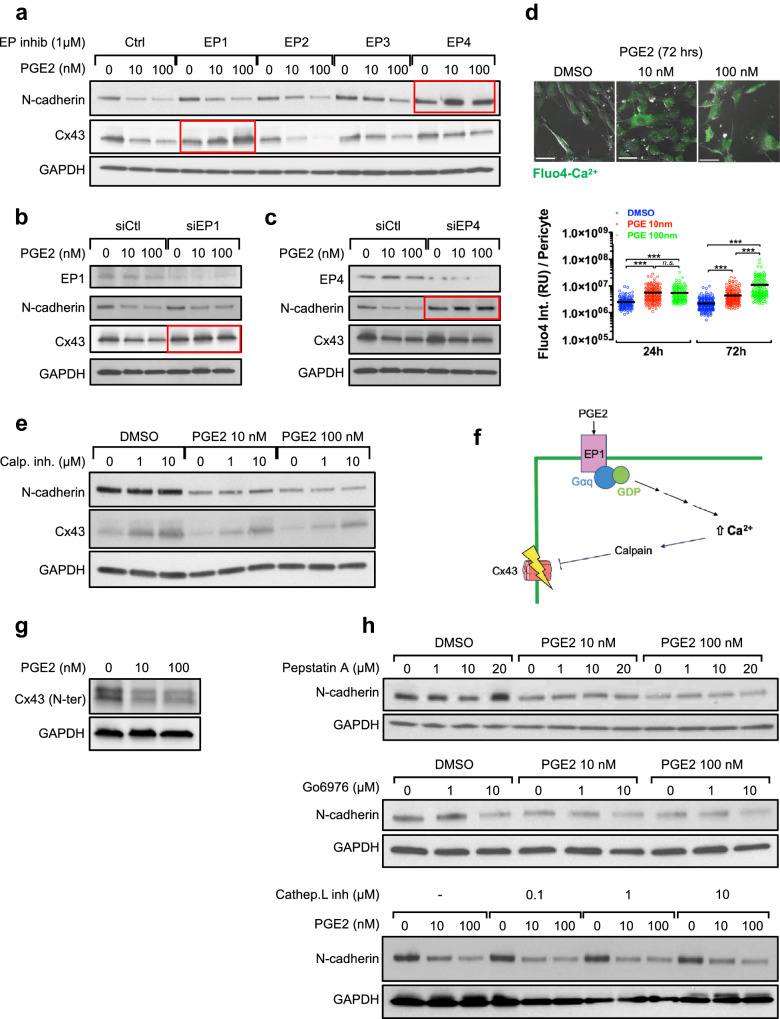
PGE2 downregulates N-cadherin via EP4 and Cx43 via EP1/Ca^2+^/calpain signaling. (**a**) HBVPs were treated with inhibitors of different EP receptors for 5 h prior to PGE2 treatment. N-cadherin and Cx43 expression was analyzed 72 h later by western blot. (**b**, **c**) EP1 or EP4 was silenced in HBVPs. Cells were treated with PGE2 24 h after siRNA transfection, and protein extracts were collected 48 h later for western blot analysis. d. The changes in intracellular calcium levels induced by PGE2 were monitored in HBVPs by Fluo4-calcium assay. One-way ANOVA test, ***p < 0.001; ns: not significant. (**e**) HBVPs were incubated 4 h with a μ-calpain inhibitor before PGE2 or DMSO treatment. N-cadherin and Cx43 expression was analyzed 72 h later by western blot. (**f**) A proposed model of Cx43 downregulation by PGE2/EP1/Ca^2+^/calpain signaling. (**g**) In order to verify that PGE2-mediated decrease of Cx43 is not a result of epitope loss following cleavage by calpain, Cx43 expression was analyzed in HBVPs following PGE2 treatment using an anti-Cx43 antibody targeting a N-terminal epitope. h. HBVPs were treated with various inhibitors of proteins suspected to be involved in PGE2-induced N-cadherin degradation. Pepstatin A was used as an inhibitor for γ-secretase and Gö6976 for PKC. The cells were treated with each inhibitor 4 h prior PGE2 treatment, and protein extracts were collected after a total of 72 h.

To elucidate which EP receptor activation by PGE2 leads to N-cadherin and Cx43 downregulation in pericytes, we used selective EP inhibitors 4 h prior to PGE2 treatment. This study demonstrated that EP4 is primarily responsible for N-cadherin downregulation while EP1 is responsible for Cx43 downregulation (Figs. 8a, S5a). The siRNA-mediated silencing of each receptor confirmed these findings (Figs. 8b,c, S5b,c).

The activation of EP1 by PGE2 results in intracellular Ca^2+^ mobilization from the endoplasmic reticulum while EP4 activation elevates intracellular cAMP. Of note, some studies reported that EP4 can either lower or raise the concentration of intracellular Ca^2+^ depending on the cellular context ^76,77^. We monitored the level of intracellular Ca^2+^ after a treatment of pericytes with PGE2 for 24 and 72 h. This study revealed a significant and sustained increase in intracellular Ca^2+^ content in pericytes following PGE2 treatment (Fig. 8d).

The elevation of cytoplasmic Ca^2+^ concentration activates calpain, an intracellular Ca^2+^-dependent cysteine protease responsible for the cleavage of various proteins including N-cadherin as previously described in cardiomyocytes, neurons and astrocytes^78,79^. To test whether the activation of calpain by the PGE2-EP1-Ca^2+^ signaling is responsible for N-cadherin and Cx43 downregulation in pericytes, we first treated pericytes with a calpain I inhibitor prior to the exposure to PGE2. This study demonstrated that the inhibition of calpain completely blocks the PGE2-mediated loss of Cx43, but not N-cadherin (Figs. 8e, S5d), suggesting that Cx43 downregulation by PGE2 is Ca^2+^-dependent (Fig. 8f). As the observed regulation of Cx43 by calpain may be due to protein cleavage, we used a N-terminal targeting antibody to corroborate the results observed with a C-terminal targeting antibody (Figs. 8g, S5e). This rules out the possibility that the apparent Cx43 downregulation reflects a loss of target epitope. The mechanism responsible for the downregulation of N-cadherin downstream of EP4 remains to be elucidated. The inhibition of potential candidates in this process, such as γ-secretase, protein kinase C and cathepsin-L, failed to restore N-cadherin upon PGE2 treatment (Fig. 8h).

### Overexpression of R-Ras fails to restore junction protein expression

**Figure 9:**
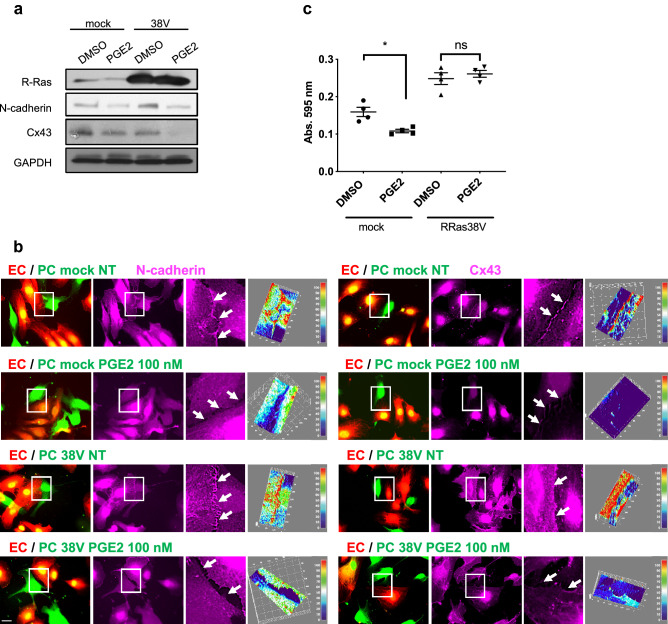
Constitutively activated R-Ras fails to restore N-cadherin and Cx43 expression upon PGE2 treatment but restores cell adhesion of pericytes. (**a**) HBVPs transduced with mock or constitutively active R-Ras38V were treated with PGE2 (100 nM) or DMSO. N-cadherin and Cx43 expression was subsequently analyzed by western blot. (**b**) Mock- and R-Ras38V-transduced HBVPs (green) were treated with PGE2 for 72 h prior to coculture with HUVECs (red) on tissue culture plates. Cells were stained for N-cadherin (left panels) and Cx43 (right panels) 18 h later. 3D-surface plots of immunofluorescence intensity are used to quantify proteins at the cell–cell interface upon PGE2 treatment. One representative picture out of at least 10 regions measured for each condition is shown. The immunoreactivity is shown with arbitrary unit. Scale bar 50 μm. (**c**) Mock- and R-Ras38V-transduced HBVPs were incubated with PGE2 (100 nM) for 72 h, detached from the cell culture dish using 0.005% trypsin and seeded in 24-well plates for 30 min in basal medium at 37 ºC. Unattached cells were removed by PBS wash, and cells were fixed and stained with a methanol/crystal violet solution. Crystal violet was subsequently extracted from attached cells to measure the absorbance at 595 nm. One-way ANOVA test, *p < 0.05; ns: not significant.

The expression of constitutively active R-Ras (R-Ras38V) upregulates N-cadherin in pericytes (Figs. 9a, S6) and promotes N-cadherin- and Cx43-mediated contacts with ECs (Fig. 9b). To determine whether R-Ras downregulation is responsible for the loss of pericyte–EC interaction by PGE2, we examined the effect of R-Ras38V expression on junctional protein expression. R-Ras38V expression did not restore N-cadherin or Cx43 expression in PGE2-treated pericytes (Fig. 9a,b, S6). However, R-Ras38V restored pericyte adhesion to culture dish, supporting that PGE2-dependent downregulation of R-Ras is responsible for weakened pericyte adhesion (Fig. 9c). Altogether, these results show that PGE2 disrupts pericytes in multiple pathways, notably through R-Ras transcriptional repression as well as junctional protein downregulation.

### Inhibition of microsomal prostaglandin E synthase-1 in colon cancer cells blocks pericyte disruption by these cells

**Figure 10:**
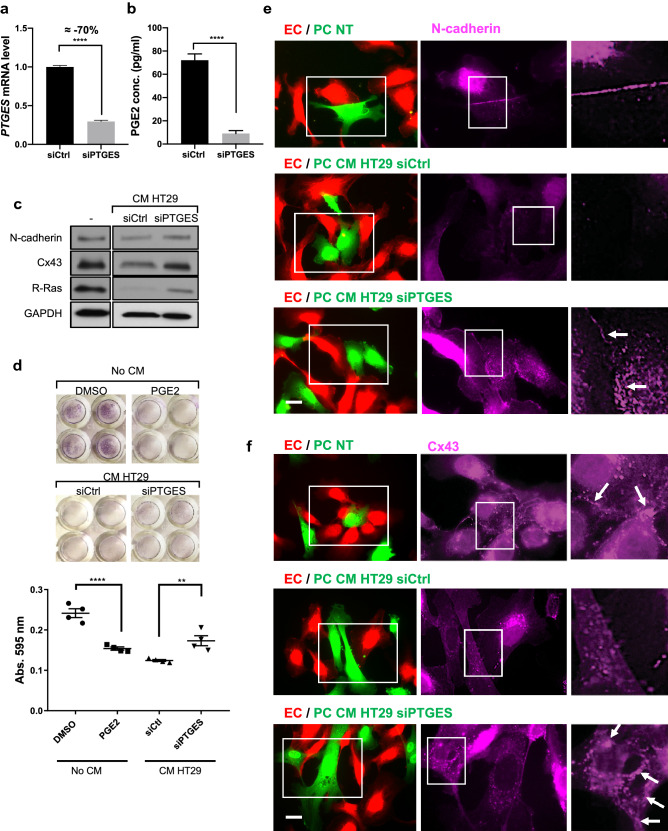
Silencing mPGES-1 prevents colon cancer cells from disrupting pericytes. HT29 human colon cancer cells were transfected with control- or *PTGES* (mPGES-1 gene)-targeting siRNA for 48 h. (**a**) Cell lysates were examined by RT-qPCR to analyze mPGES-1 expression in HT29 cells. The *PTGES* mRNA level was normalized to cyclophilin A. Fold change relative to the control is shown. (**b**) ELISA was performed to measure the concentration of PGE2 in the conditioned media. Student *t*-test, ****p < 0.0001. (**c**) HBVPs were incubated either with fresh culture medium as a control condition (–) or with conditioned medium (CM) from control (siCtrl) or *PTGES*-silenced HT29 cells. Western blot was performed to analyze N-cadherin, Cx43 and R-Ras expression 48 h later. (**d**) HBVPs were incubated with DMSO or PGE2 (100 nM) for 72 h, or treated with conditioned medium from control- or *PTGES*-silenced HT29 cells for 48 h. The cells were detached, and the adhesion of these cells to new culture plate was determined by crystal violet staining and absorbance at 595 nm. The data shown is a representative of three independent experiments performed in quadruplicates. One-way ANOVA test, **p < 0.01; ****p < 0.0001. (**e**, **f**) HBVPs (green) were incubated either with fresh culture medium as a control, or with conditioned medium from control- or *PTGES*-silenced HT29 cells for 48 h prior to coculturing with HUVECs (red) on culture plates. Cells were stained for N-cadherin (**e**) or Cx43 (**f**) 18 h later. Scale bar 50 μm.

The role of PGE2 as a tumor-promoting factor is well-documented for various types of cancers. In colorectal cancer, for example, both cancer cells and stromal cells express COX-2 and produce PGE2 contributing to the malignant progression^80,81^. It is conceivable that high PGE2 concentration in the tumor microenvironment disrupts tumor vessels by disrupting pericyte–EC interaction, thereby contributing to tumor invasion, metastasis and poor drug delivery, as well as weak anti-tumor immune response. To understand the role of cancer cell-derived PGE2, we treated pericytes with conditioned media from HT29 human colon adenocarcinoma. Microsomal prostaglandin E synthase-1 (mPGES-1) is an enzyme responsible for the biosynthesis of PGE2 downstream of COX1/COX2. HT29 cells with or without mPGES-1 silencing were used to prepare conditioned media. The mPGES-1 silencing (Fig. 10a) resulted in a drastic decrease in the PGE2 concentration in the conditioned media as shown by ELISA (Fig. 10b). The pericytes incubated for 48 h with the control conditioned media displayed reductions of N-cadherin, Cx43, and R-Ras expression compared to untreated pericytes (Fig. 10c, S7). The effect of the conditioned media was attenuated by mPGES-1 silencing in HT29 cells to suppress PGE2 production. Furthermore, mPGES-1 silencing in HT29 cells partially rescued pericyte adhesion (Fig. 10d) and restored pericyte–EC interaction in 2-D coculture as shown by N-cadherin and Cx43 accumulation at the cell–cell junctions (Fig. 10e,f). These results suggest that the colon cancer cells limit the ability of pericytes to interact with ECs in part via production of PGE2.

## Discussion

**Figure 11:**
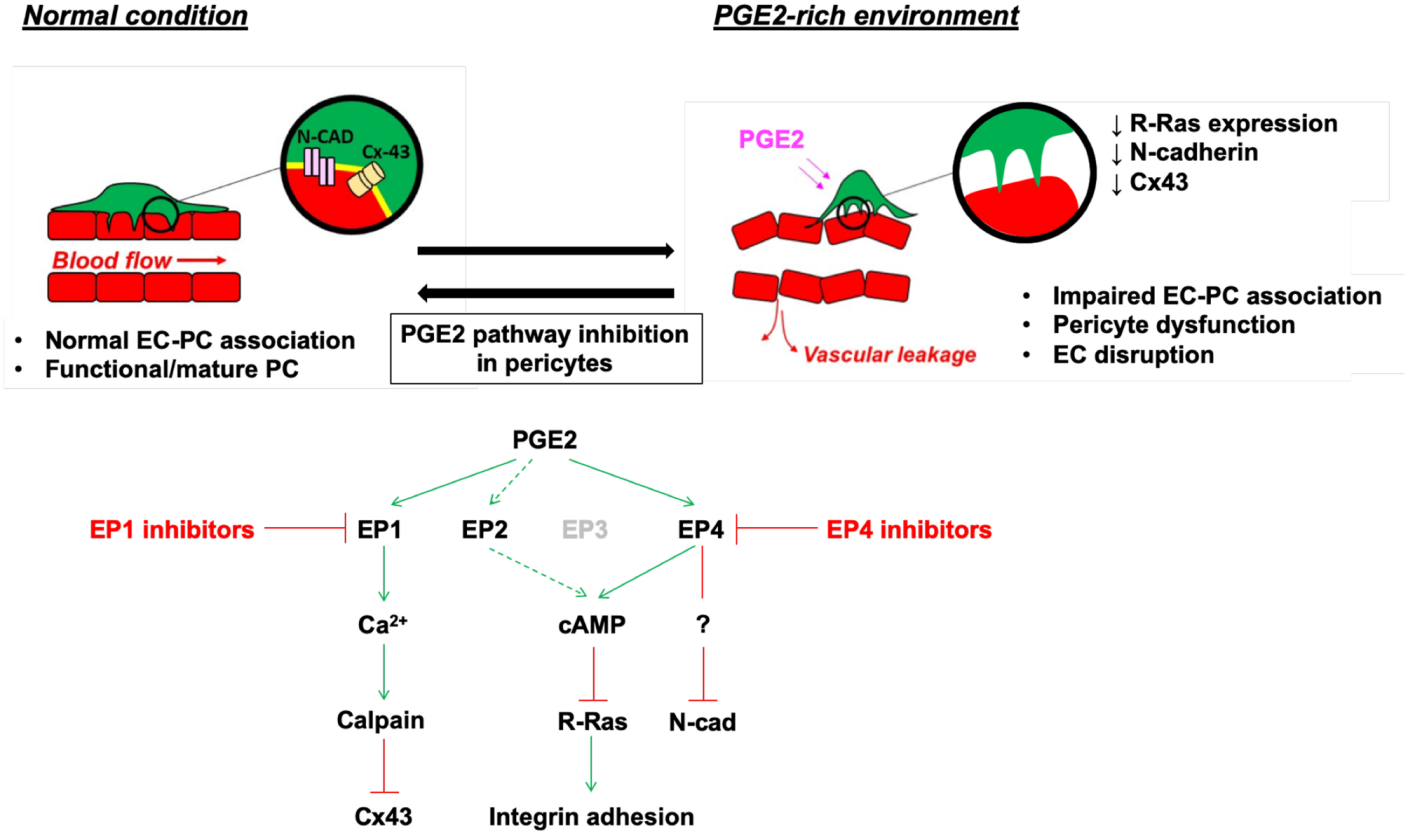
Exposure to PGE2 disrupts pericytes and damages vessel wall integrity. In normal condition, pericytes are closely interacting and communicating with ECs via adherens junctions (N-cadherin), gap junctions (Cx43), and cell adhesion to basement membrane extracellular matrix (yellow). The exposure to PGE2-high environment in pathological conditions, such as colon cancer, disrupts normal pericyte function, which in turn causes disruption of the EC lining of blood vessel wall. The use of EP1 and EP4 inhibitors can restore the vascular damages induced by PGE2.

Elevated expression of COX2 and concomitant overproduction of PGE2 are frequently observed in a variety of malignancies. In the tumor microenvironment, PGE2 acts as an immunosuppressor^38,82,83^ and promotes angiogenesis and hyperpermeability of the tumor vasculature^38,84–86^. The importance of pericytes in vascular development and stability is well established. Although many studies have focused on the effects of PGE2 on cancer cells, immune cells, and ECs, to our knowledge, no previous study has examined the direct effect of PGE2 on pericytes and its impact on blood vessels. In this study, we demonstrated significant disruptive effects of PGE2 on pericytes and their interaction with ECs (Fig. 11). We showed that PGE2 induces a phenotypic change of pericytes to assume dendritic morphology with loose adhesion to the ECM. The reduced cell adhesion coincides with a marked decrease in the expression of proteins that regulate cell adhesion, such as FAK and paxillin as well as R-Ras, which reinforces cell adhesion to the ECM via integrin activation^36^. The observed R-Ras downregulation is also consistent with the disruption of pericyte–EC interaction^31^. Recently, we reported that a prolonged intracellular cAMP elevation in ECs ultimately increases vascular permeability through transcriptional repression of *RRAS* and the loss of VE-cadherin at adherens junctions^59^. PGE2 exerts this effect on ECs via EP4-dependent cAMP signaling^59^. In the current study, we showed that EP4 is also responsible for the *RRAS* repression in pericytes. Since cAMP signaling is the major pathway for EP4-dependent PGE2 signaling, our finding underscores the role of cAMP in vascular instability.

The inability of PGE2-exposed pericytes to make close interaction with ECs is also attributed to markedly decreased N-cadherin and Cx43 at adherens junctions and gap junctions that mediate the communication between the two cell types. The N-cadherin is crucial for stabilizing the interaction between the two cell types, and blocking or loss of N-cadherin results in decreased pericyte association with the endothelium^87–89^. Cx43-dependent gap junction is required for the quiescence of ECs and vessel stabilization, and Cx43 downregulation reduces pericyte coverage and enhances tumor angiogenesis in murine mammary tumors^90^. In our study, we showed that Cx43 in the pericyte–EC gap junction is downregulated by calpain in pericytes via EP1/Ca^2+^ signaling induced by PGE2. As for the N-cadherin downregulation by PGE2, we provided evidence that the mechanism is post-transcriptional and EP4-dependent. However, the downstream effectors of this mechanism remain to be determined.

High levels of PGE2 are often found in human cancer. Colorectal carcinoma cells are among the tumor cells that produce high amount of PGE2 due to the aberrant expression of COX-2 and mPGES-1^80,81^. We demonstrated that conditioned media from HT29 colon cancer cells strongly downregulates R-Ras and Cx43, and to a lesser extent, N-cadherin. Pericyte adhesion and pericyte–EC interaction were both impaired when pericyte are pre-exposed to the HT29 conditioned media. These disruptive effects of HT29 conditioned media on pericytes were significantly attenuated when mPGES-1 was silenced in HT29 cells. Thus, colon cancer-derived factors interfere with normal function of pericytes, and PGE2 is the major mediator of such effects.

Since pericyte dysfunction contributes to tumor malignancy in multiple ways, a therapeutic strategy to stabilize pericytes may be a potential approach for cancer treatment. Moreover, the restoration of intimate interaction between pericytes and ECs is expected to have a vascular normalization effect, resulting in improved drug delivery and sensitization of tumor cells to radiation as well as enhanced anti-tumor immunity^31,91,92^. We demonstrated how detrimental the PGE2-rich environment is to the normal function of pericytes and their interaction with ECs. These findings suggest that the blockade of PGE2 pathway may offer an effective way for pericyte “normalization” in cancer. Another potential therapeutic application of PGE2 blockade may be for the cerebral vasculature affected by PGE2. Pericyte–EC interaction in cerebral microvasculature is compromised in Alzheimer’s disease^16,17^. The blood–brain-barrier (BBB) breakdown due to the loss of pericytes is thought to accelerate neurodegeneration in Alzheimer’s disease^16,19,21^. PGE2 blockade may be useful for preventing or delaying Alzheimer’s disease progression.

COX inhibitors have been shown to prevent, delay, or reduce tumor growth^93^. The use of these drugs also appears to be linked to delayed onset or progression of Alzheimer’s disease^94^. However, these drugs also inhibit the production of other prostanoids such as prostacyclin. Therefore, associated cardiovascular and gastrointestinal adverse effects limit the effective use of the conventional COX inhibitors. Alternative strategies are being developed to specifically inhibit the PGE2 pathway. For instance, mPGES-1 is a promising target because mPGES-1 inhibition only impedes PGE_2_ production without affecting the production of other prostanoids^95,96^. Two selective inhibitors of mPGES-1, GRC 27,864 (Glenmark Pharmaceuticals) and LY3023703 (Eli Lilly and Company), are currently under clinical trials^97,98^. Moreover, selective inhibitors of each EP receptor subtype have been developed to specifically block the effects of PGE2^66,68,99^. Pericyte stabilization may be an important mechanism of action of these emerging drugs in the interventions of cancer and Alzheimer’s disease.

## Supplementary information

Supplementary file1 (PDF 39147 kb)

## Data Availability

The datasets and detailed experimental protocols performed and analyzed in the present study are available from the corresponding author upon request.
