# Supplementary material for: Prostaglandin E2 breaks down pericyte–endothelial cell interaction via EP1 and EP4-dependent downregulation of pericyte N-cadherin, connexin-43, and R-Ras
Source: Sci Rep. 2020 Jul 7;10:11186. doi: 10.1038/s41598-020-68019-w (PMC7341885; doi:10.1038/s41598-020-68019-w)
Supplement: Supplementary file 1 — Supplementary file1 (PDF 39147 kb) [file 41598_2020_68019_MOESM1_ESM.pdf]

Prostaglandin E2 breaks down pericyte-endothelial cell interaction via EP1 and EP4-dependent downregulation of pericyte N-cadherin, connexin-43, and R-Ras

Carole Y. Perrot, Jose L. Herrera, Ashley E. Fournier-Goss, Masanobu Komatsu*

Supplemental material and methods

Gel contraction assay

First, the optimal concentration of NaOH to add to the rat tail collagen I (1 mg/ml)/media mixture was determined to optimize gel solidification (detailed process available upon request). The titration of collagen indicated a concentration of NaOH 10mM. Pericytes treated with PGE2 (10 or 100 nM) or DMSO for 72 hours were trypsinized, resuspended in complete pericyte medium, and mixed with collagen and NaOH to a final concentration of 80,000 cells/ml, 1mg/ml collagen, and 10 mM NaOH. The mixture was supplemented with PGE2 or DMSO and immediately poured into 24 well-plates (500 µl/well) to solidify at room temperature for 20 min. Gels were covered with 500 µl of complete pericyte medium and gently dissociated from the mold by carefully inserting a 200 µl-pipet tip along gel edges, then incubated at 37°C. Gels were photographed 24 hours later using a Nikon SMZ1270 microscope and NIS Elements BR5.20.01 software. Cell contraction was analyzed using ImageJ by measuring the ratio between gel area and an average of 3 cell-free gels used as reference. The experiment was repeated twice using four technical replicates.

Supplementary Figure 1. a. Human brain microvascular pericytes (HBVPs) were treated with increasing concentrations of PGE2 for 72 hours, then stained for nuclei (DAPI, blue) and actin (red). Following the treatment, HBVPs display smaller cell bodies and develop numerous thin dendrite-like projections (white arrows). b. HBVP contractility was assessed by performing a gel contraction assay following a 72-hour exposure to PGE2. Gel area was measured 24 hours after cell seeding. Results are displayed as a ratio between contracted gel areas and cell-free, uncontracted gel areas from two independent experiments performed in quadruplicates. c. The levels of phospho-myosin light chain (P-MLC2) and total MLC2 in HBVPs were analyzed by Western blot analysis at different time points post-PGE2 treatment and quantified. d. Quantification of immunoblots from Figure 1g. Results represents ratios between phosphorylated versus total protein levels of Src, FAK and Paxillin. One-way ANOVA, *$p<0.05$; **$p<0.01$; ***$p<0.001$; ****$p<0.0001$; ns, not significant.

Supplementary Figure 2. a. The decrease of phospho-FAK (green) in pericytes following PGE2 treatment was confirmed by immunostaining (actin in red, DAPI in blue). Scale bar, 25 μm. b. HBVPs were treated with either control DMSO or forskolin (FSK, 10 μM) for 72 hours before protein extraction. Western blot analysis was performed to assess the expression of proteins involved in cell adhesion: Src/phospho-Src, FAK/phospho-FAK, and paxillin/phospho-paxillin. GAPDH was used as a loading control. Immunoblots were quantified and results represent the ratio between phosphorylated versus total protein levels of Src, FAK, or Paxillin. Student *t*-test, *$p<0.05$; ****$p<0.0001$; ns, not significant. c. HBVPs were stained for P-CREB (green) following a 72-hour PGE2 treatment. The increased intensity of nuclear staining demonstrates the activation of CREB transcriptional activity by cAMP signaling. Scale bar: 25 μm. P-CREB staining was quantified as a ratio between the nuclear fluorescence intensity and nuclei area. Between 60 and 80 nuclei from 5 distinct pictures were analyzed for each condition. One-way ANOVA, *$p<0.05$; ****$p<0.0001$ d. 3-D culture of CellTracker Red-stained HUVECs on Matrigel (18 hours post-cell seeding). Scale bar, 500 μm.

Supplementary Figure 3. Quantification of immunoblots from Figure 5. a. Analysis of R-Ras protein expression in HVBPs following a 48-hour PGE2 treatment. One-way ANOVA, *$p<0.05$. b, c. Analysis of R-Ras expression in PGE2-treated HBVPs co-treated with EP1-4

inhibitors (b) or transfected with control or EP4-targeting siRNA (c). The results represent a ratio between R-Ras and GAPDH protein levels and reflect the mean quantification ± SEM of two or three experiments. Two-way ANOVA, **$p<0.01$; ***$p<0.001$; ns, not significant.

Supplementary Figure 4. Quantification of immunoblots from Figure 7. Analysis of N-cadherin and Cx43 protein expression in HVBPs following a 48-hour PGE2 treatment. The results represent a ratio between N-cadherin or Cx43 and GAPDH protein levels and reflect the mean quantification ± SEM of three experiments. One-way ANOVA, *$p<0.05$.

Supplementary Figure 5. Quantification of immunoblots from Figure 8. a, b, c. Analysis of N-cadherin and Cx43 expression in HBVPs co-treated with PGE2 and EP1-4 inhibitors (a) or transfected with control, EP1 (b) or EP4-targeting siRNA (c). Two-way ANOVA, *$p<0.05$; **$p<0.01$; ***$p<0.001$; ns, not significant. d. Cx43 protein level was measured in PGE2-treated HBVPs in response to calpain inhibition. e. Analysis of Cx43 expression in HBVPs following PGE2 exposure using a N-terminal CX43-targeting antibody. Quantification results represent a ratio between N-cadherin or Cx43 and GAPDH protein levels and display means ± SEM of two or three experiments.

Supplementary Figure 6. Quantification of immunoblots from Figure 9. Analysis of N-cadherin and Cx43 expression in mock- and R-Ras38V-transduced HBVPs with/without PGE2 treatment. The graphs represent a ratio between N-cadherin or Cx43 and GAPDH protein levels and display means ± SEM of three experiments. Two-way ANOVA, ns, not significant.

Supplementary Figure 7. Quantification of immunoblots from Figure 10. The expression of N-cadherin, Cx43 and R-Ras was analyzed in HBVPs either untreated or prealably incubated with conditioned medium from control- *vs*. PTGES-siRNA-transfected HT29 cells. Quantification results are calculated as a ratio between N-cadherin, Cx43 or R-Ras and GAPDH protein levels and represent means ± SEM of three experiments. One-way ANOVA, *$p<0.05$; **$p<0.01$.

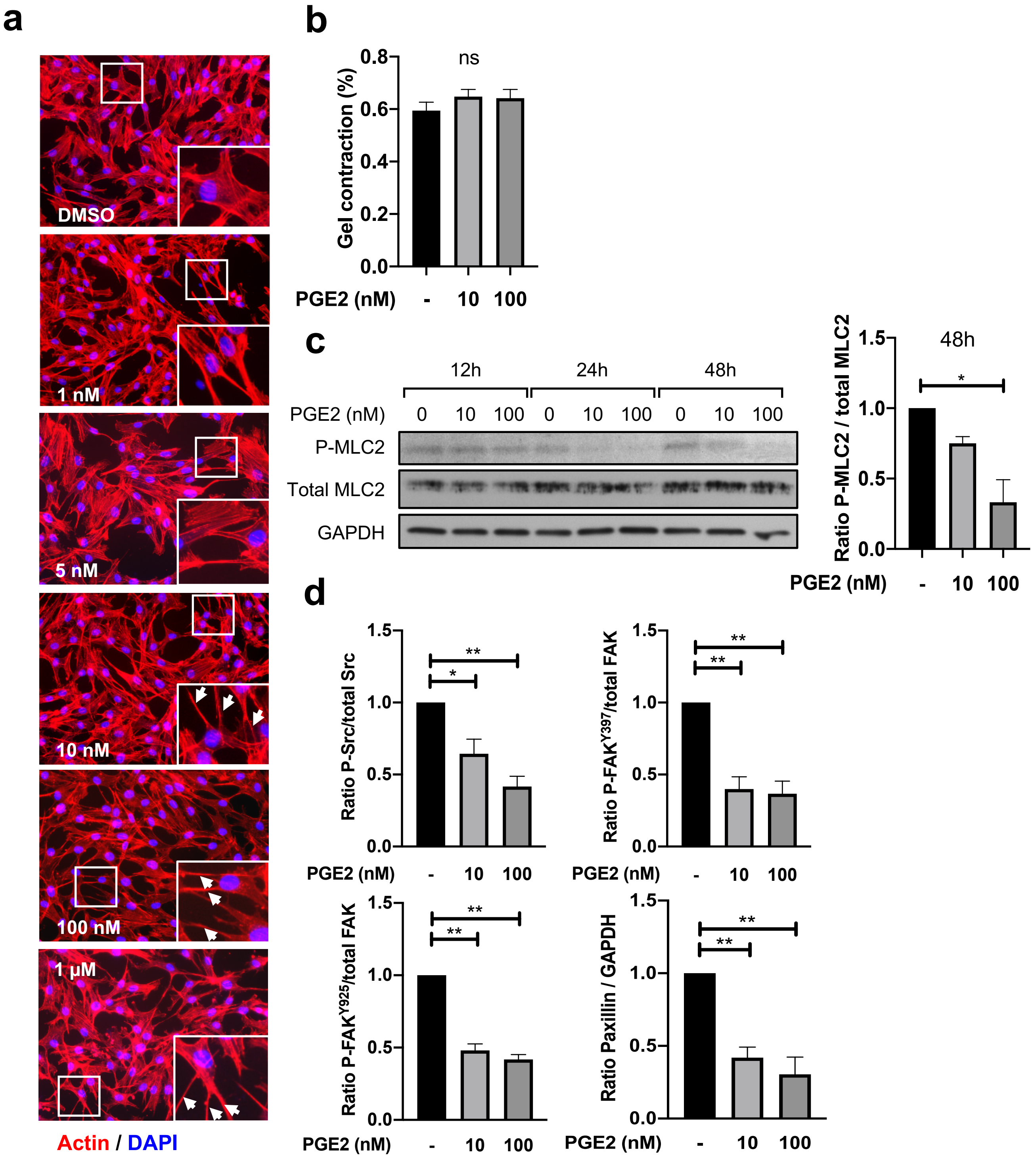

Supplementary figure 1

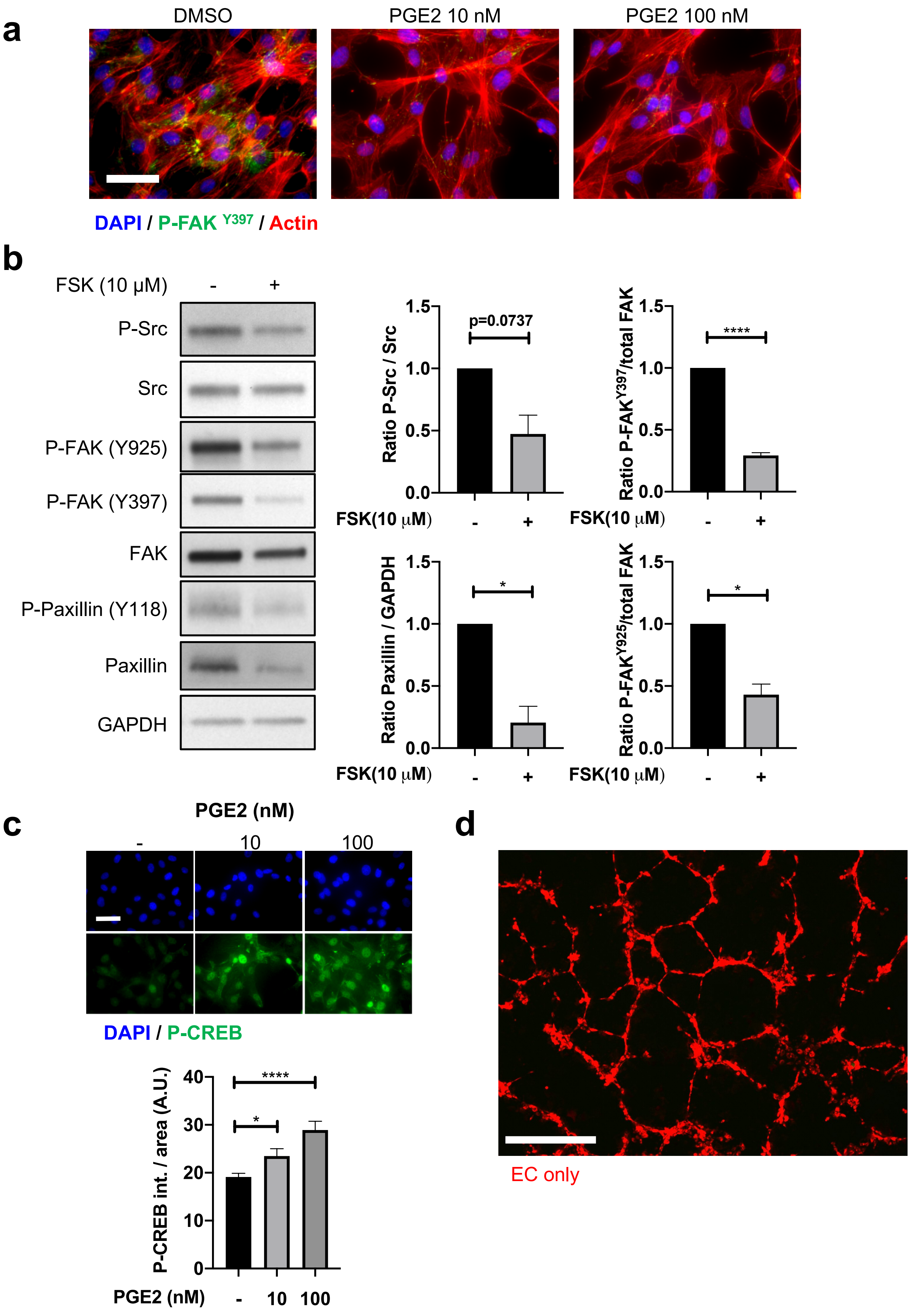

Supplementary figure 2

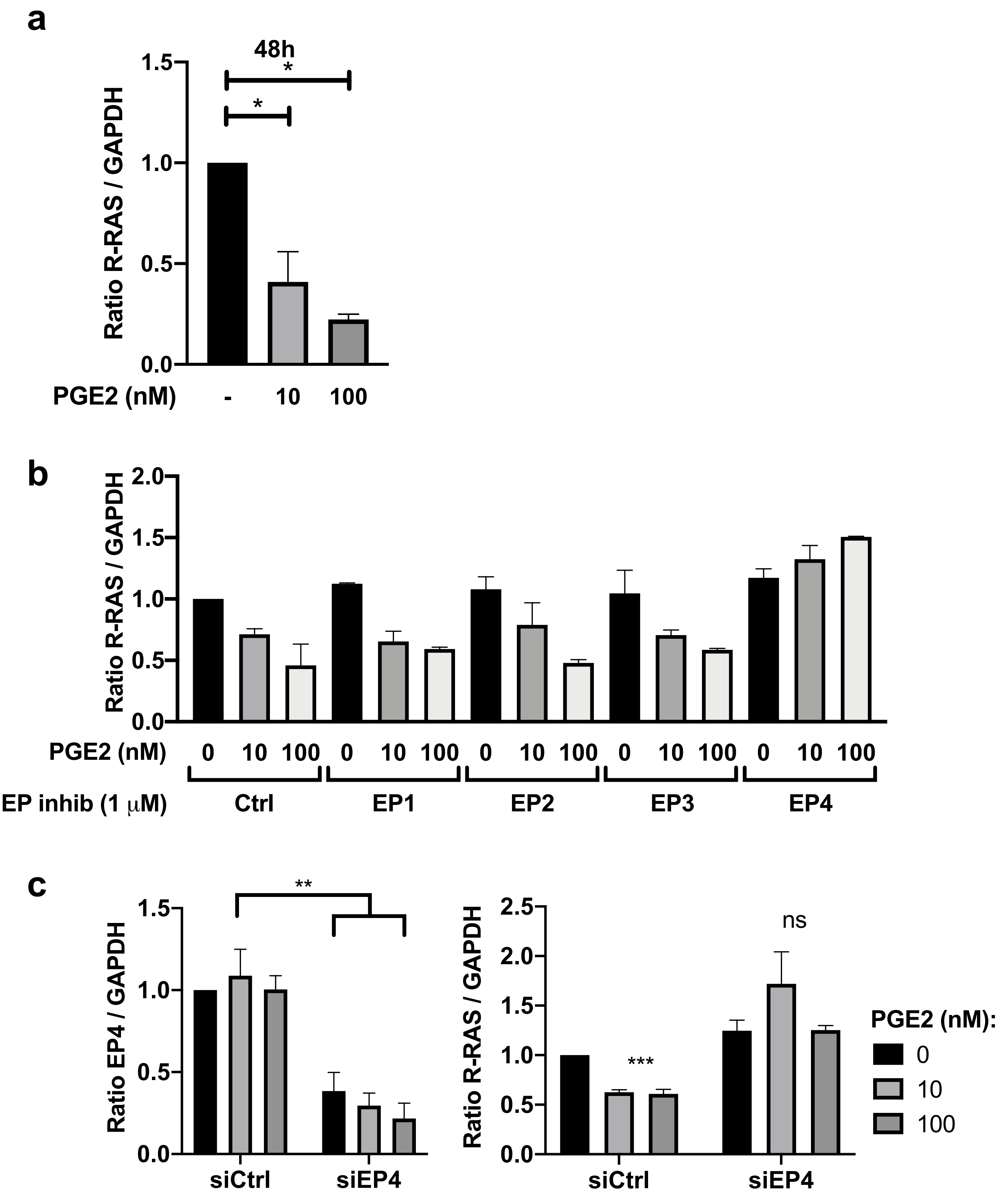

Supplementary figure 3

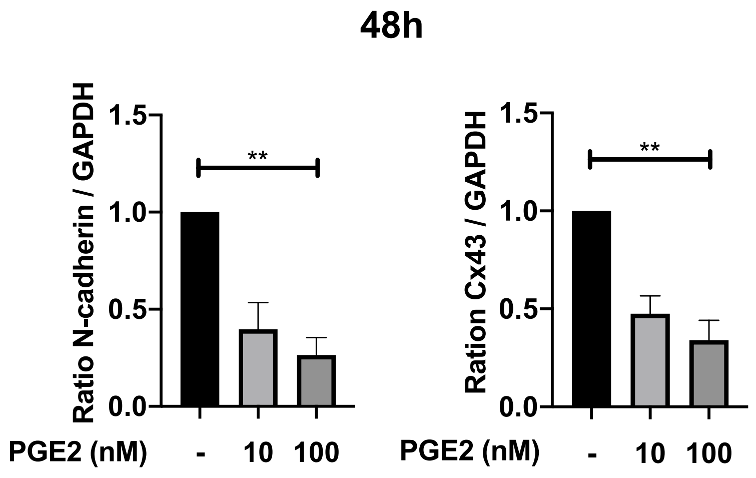

Supplementary figure 4

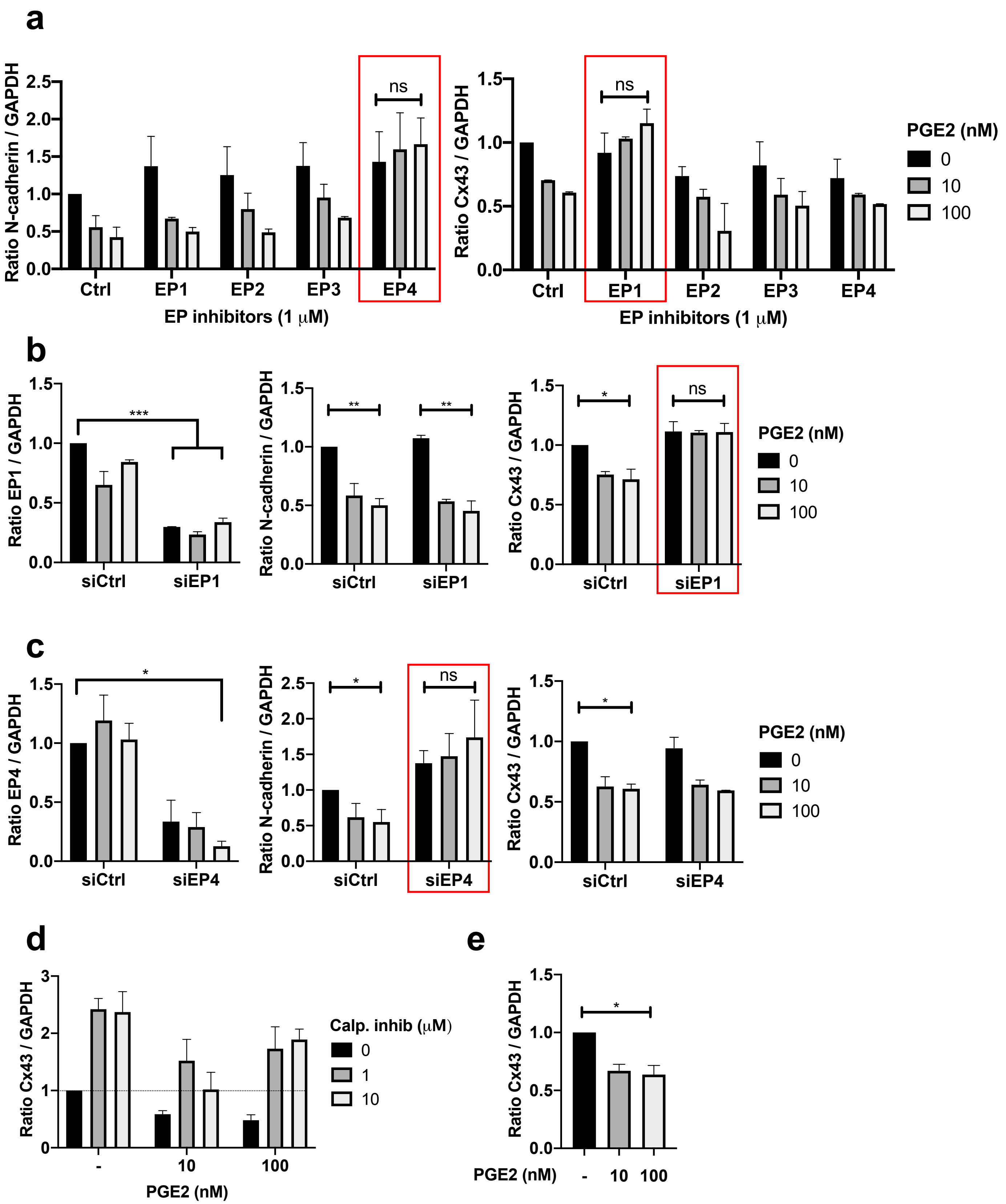

Supplementary figure 5

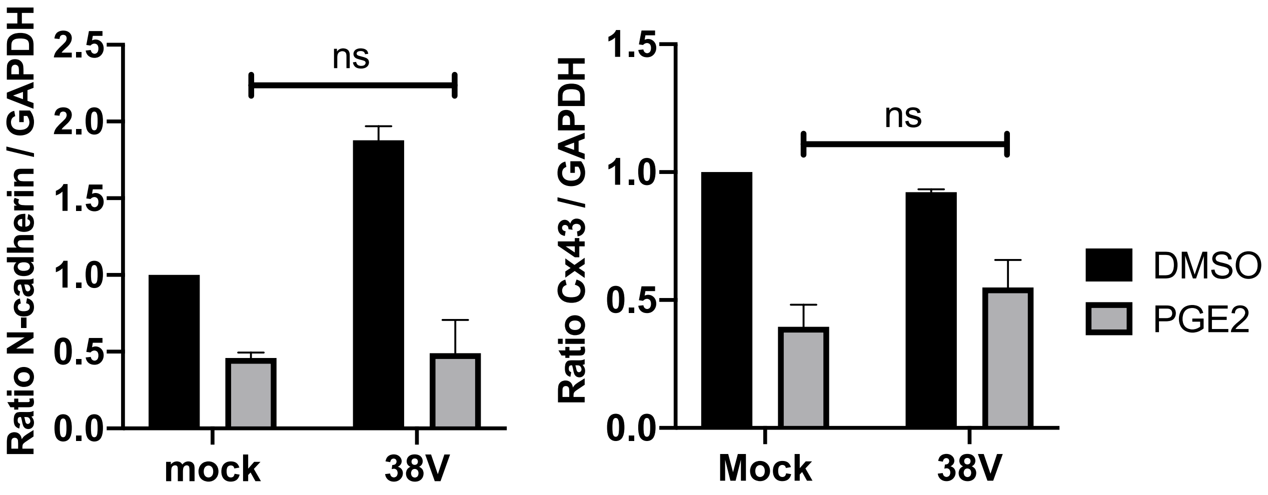

Supplementary figure 6

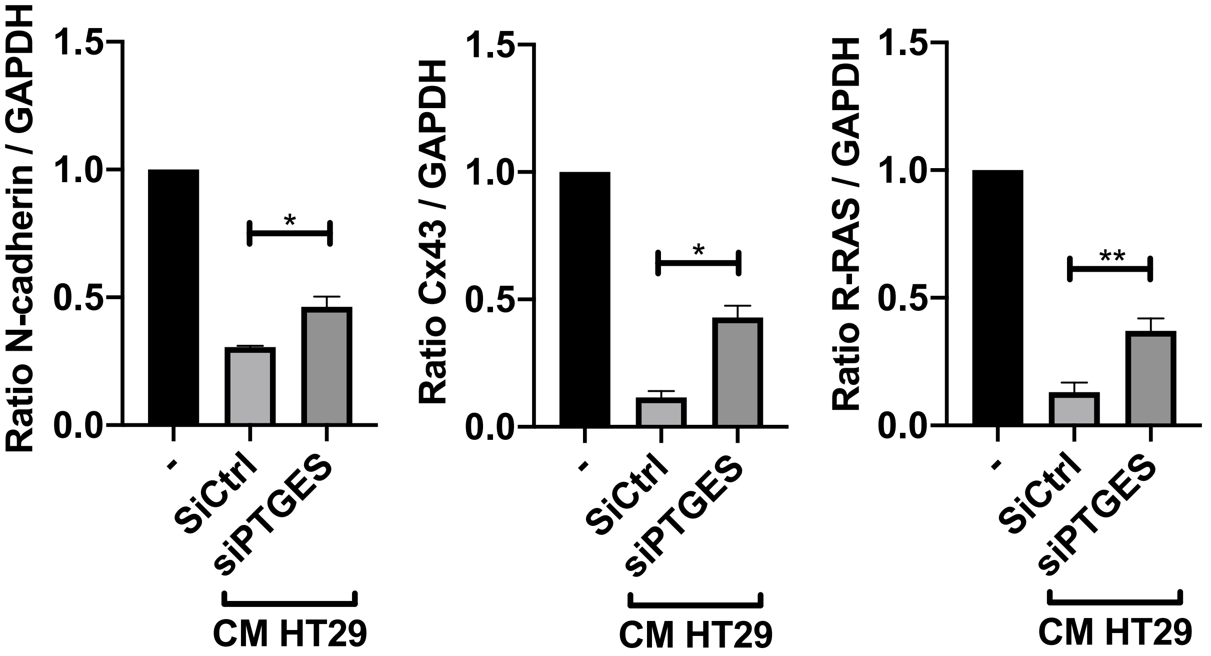

Supplementary figure 7

Prostaglandin E2 breaks down pericyte-endothelial cell interaction via EP1 and EP4-dependent downregulation of pericyte N-cadherin, connexin-43, and R-Ras

Carole Y. Perrot, Jose L. Herrera, Ashley E. Fournier-Goss, Masanobu Komatsu*

SUPPLEMENTAL INFORMATION

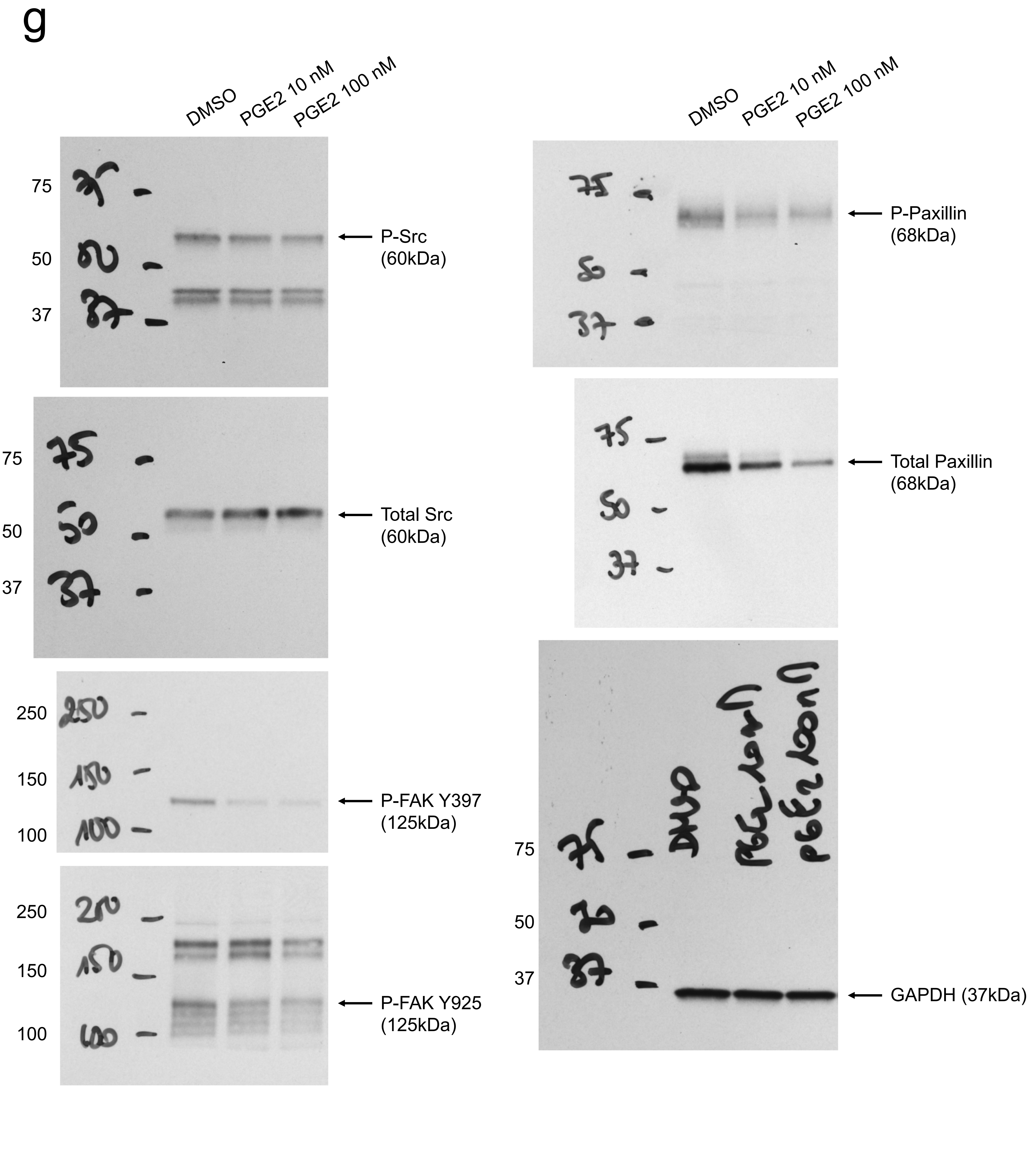
g
DMSO
PGE2 10 nM
PGE2 100 nM
75
50
37
P-Src
(60kDa)
Total Src
(60kDa)
250
150
100
P-FAK Y397
(125kDa)
P-FAK Y925
(125kDa)
P-Paxillin
(68kDa)
Total Paxillin
(68kDa)
GAPDH (37kDa)

Figure 1

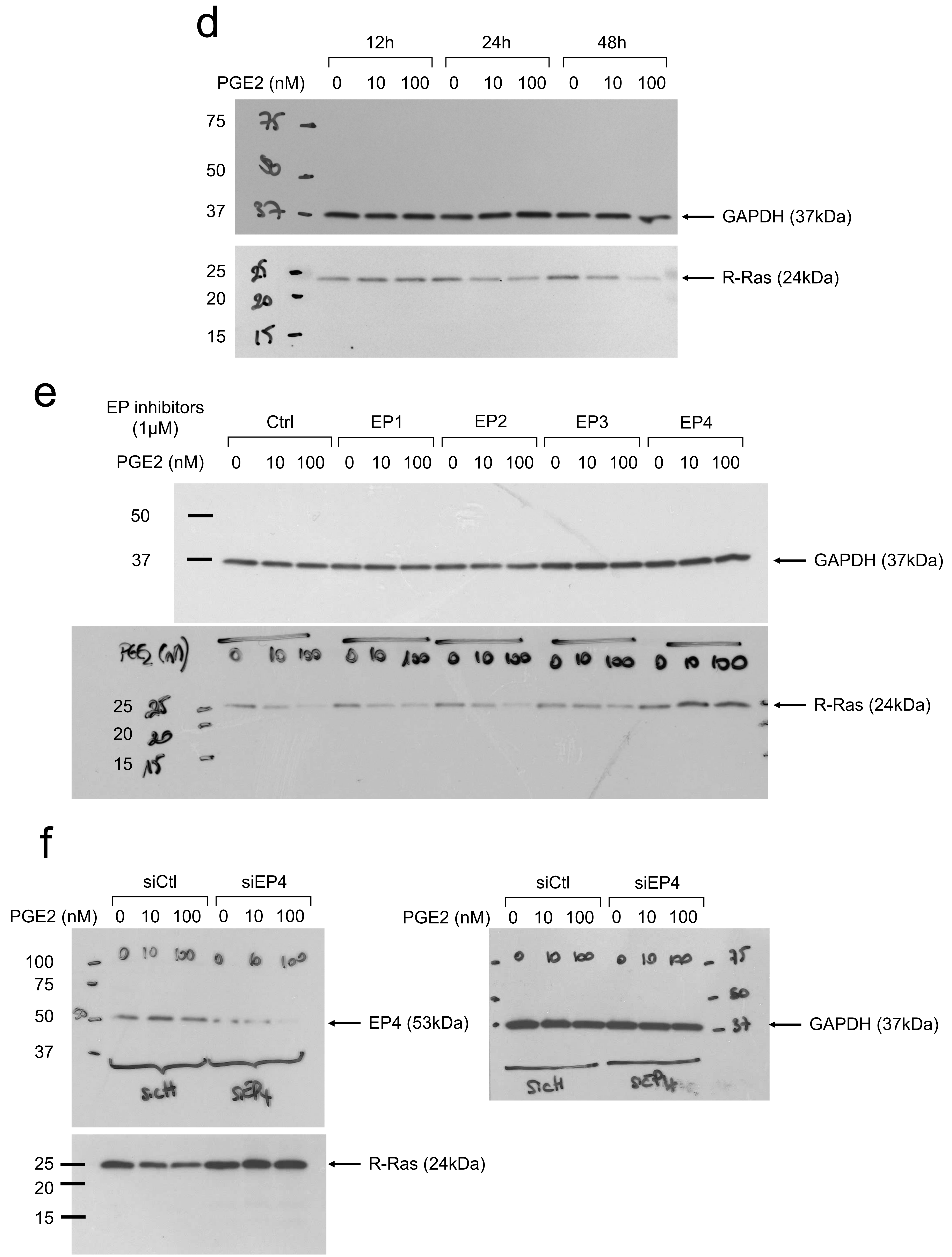
d
12h
24h
48h
PGE2 (nM)
0 10 100 0 10 100 0 10 100
75
50
37
GAPDH (37kDa)
25
20
15
R-Ras (24kDa)
e
EP inhibitors (1μM)
Ctrl
EP1
EP2
EP3
EP4
PGE2 (nM)
0 10 100 0 10 100 0 10 100 0 10 100 0 10 100
50
37
GAPDH (37kDa)
25
20
15
R-Ras (24kDa)
f
siCtl
siEP4
PGE2 (nM)
0 10 100 0 10 100
100
75
50
37
EP4 (53kDa)
25
20
15
R-Ras (24kDa)
siCtl
siEP4
PGE2 (nM)
0 10 100 0 10 100
GAPDH (37kDa)

Figure 5

a

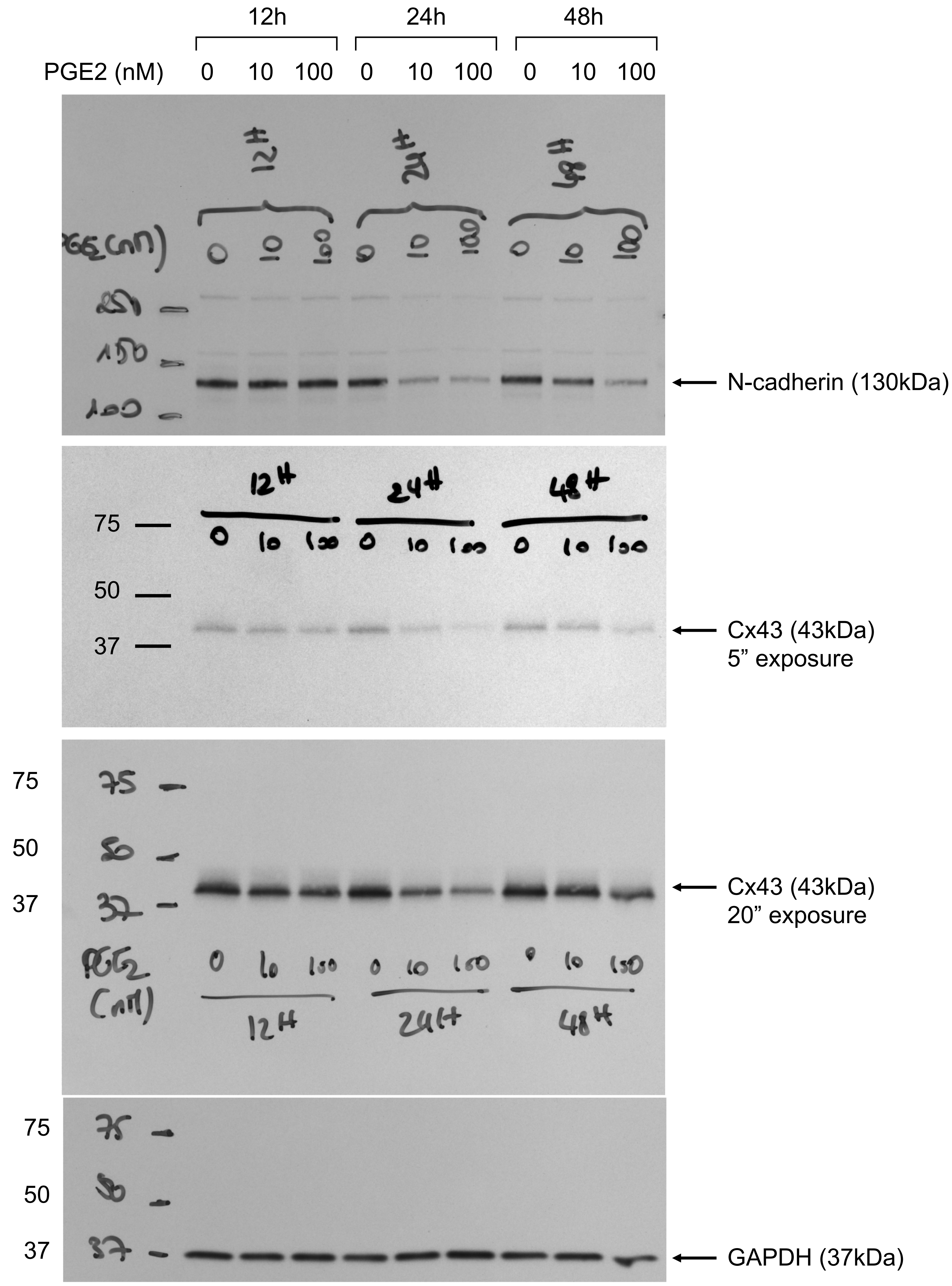
12h
24h
48h
PGE2 (nM)
0
10
100
0
10
100
0
10
100
N-cadherin (130kDa)
75
50
37
Cx43 (43kDa)
5” exposure
75
50
37
Cx43 (43kDa)
20” exposure
75
50
37
GAPDH (37kDa)

Figure 7

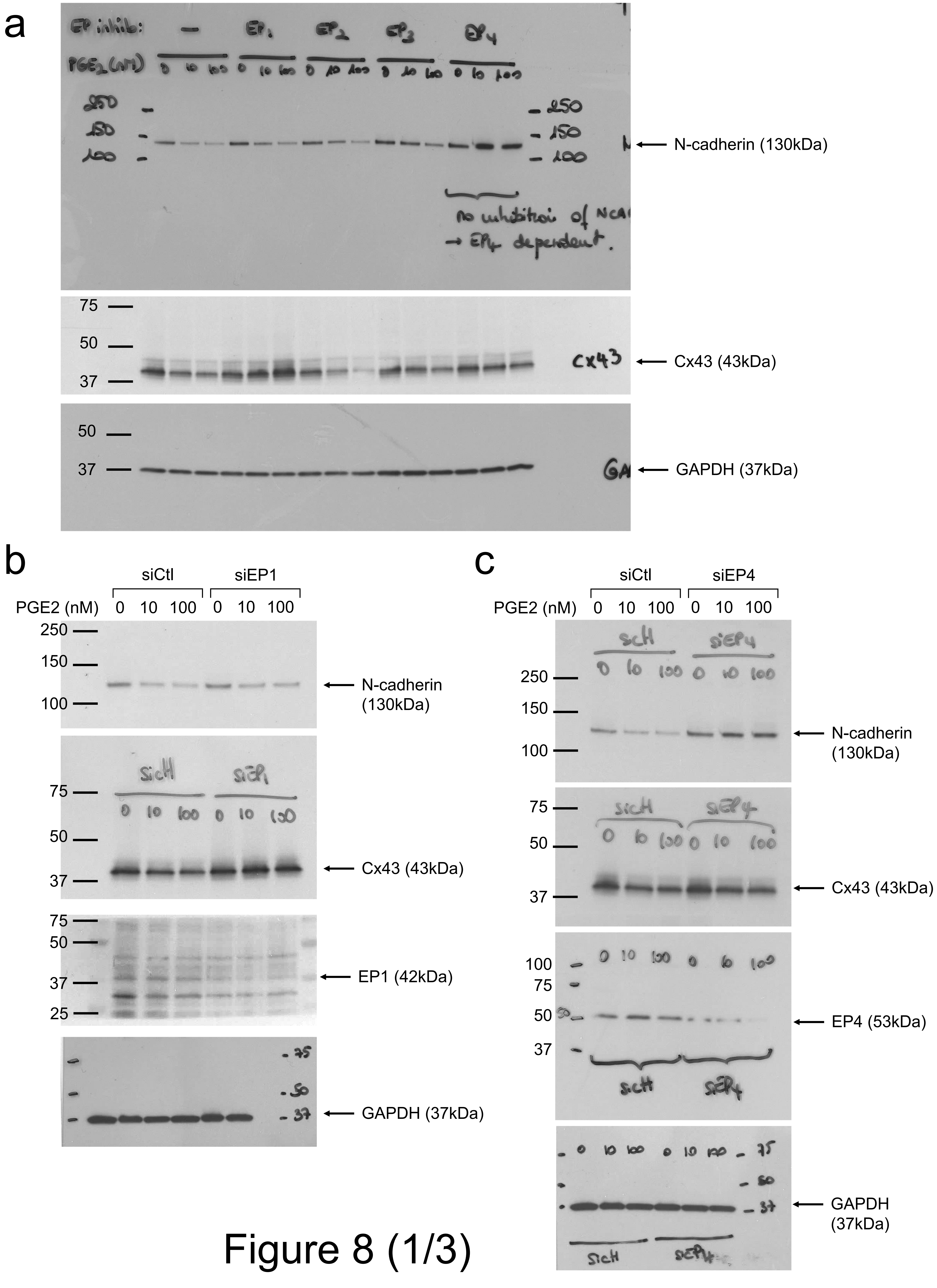
a
N-cadherin (130kDa)
Cx43 (43kDa)
GAPDH (37kDa)
75
50
37
b
siCtl
siEP1
PGE2 (nM)
0 10 100 0 10 100
250
150
100
N-cadherin (130kDa)
Cx43 (43kDa)
EP1 (42kDa)
25
GAPDH (37kDa)
c
siCtl
siEP4
EP4 (53kDa)

Figure 8 (1/3)

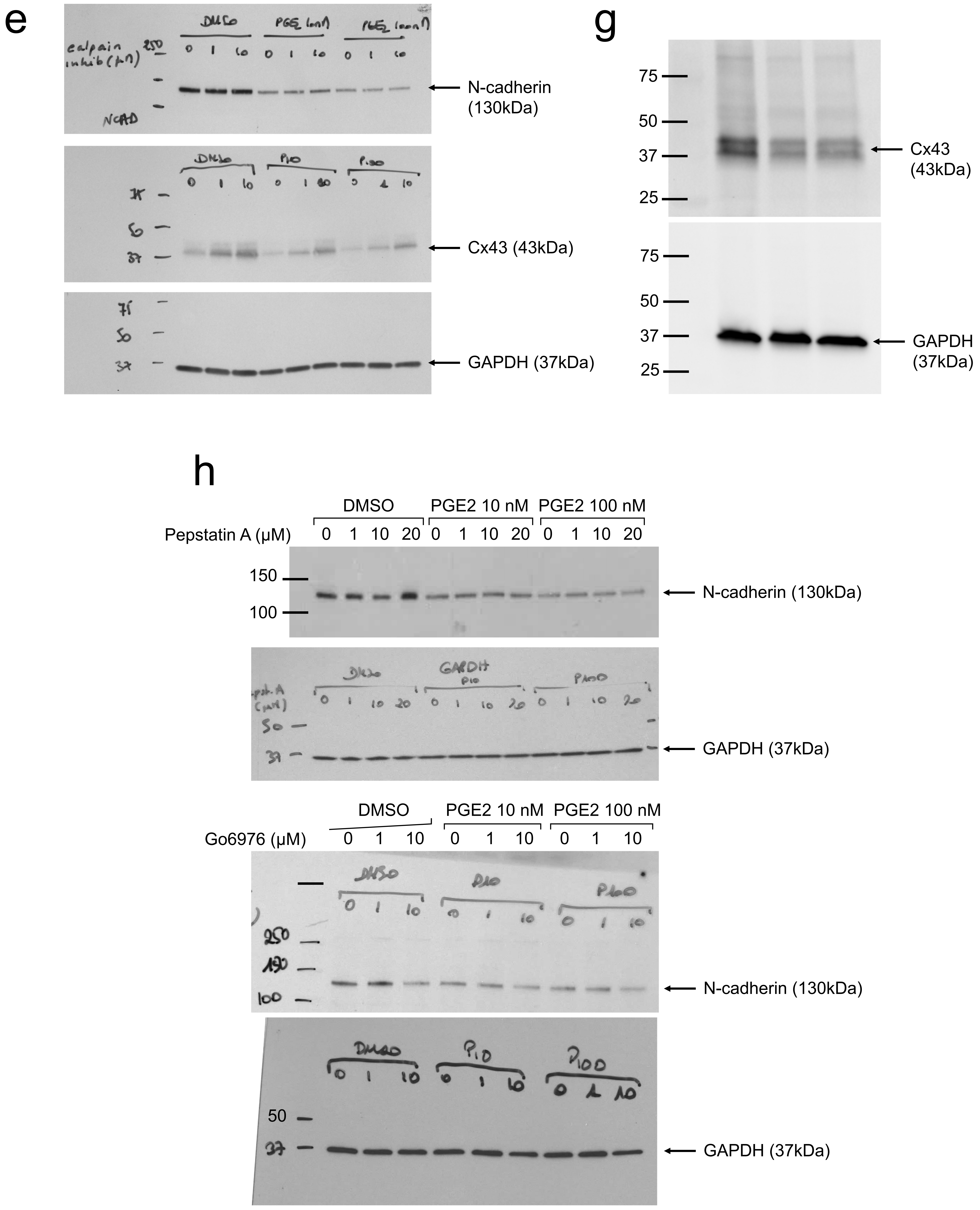
e
N-cadherin (130kDa)
Cx43 (43kDa)
GAPDH (37kDa)
g
75
50
37
25
Cx43 (43kDa)
GAPDH (37kDa)
h
DMSO
PGE2 10 nM
PGE2 100 nM
Pepstatin A (µM)
0 1 10 20 0 1 10 20 0 1 10 20
150
100
N-cadherin (130kDa)
GAPDH (37kDa)
Go6976 (µM)
0 1 10 0 1 10 0 1 10
N-cadherin (130kDa)
50
GAPDH (37kDa)

Figure 8 (2/3)

g

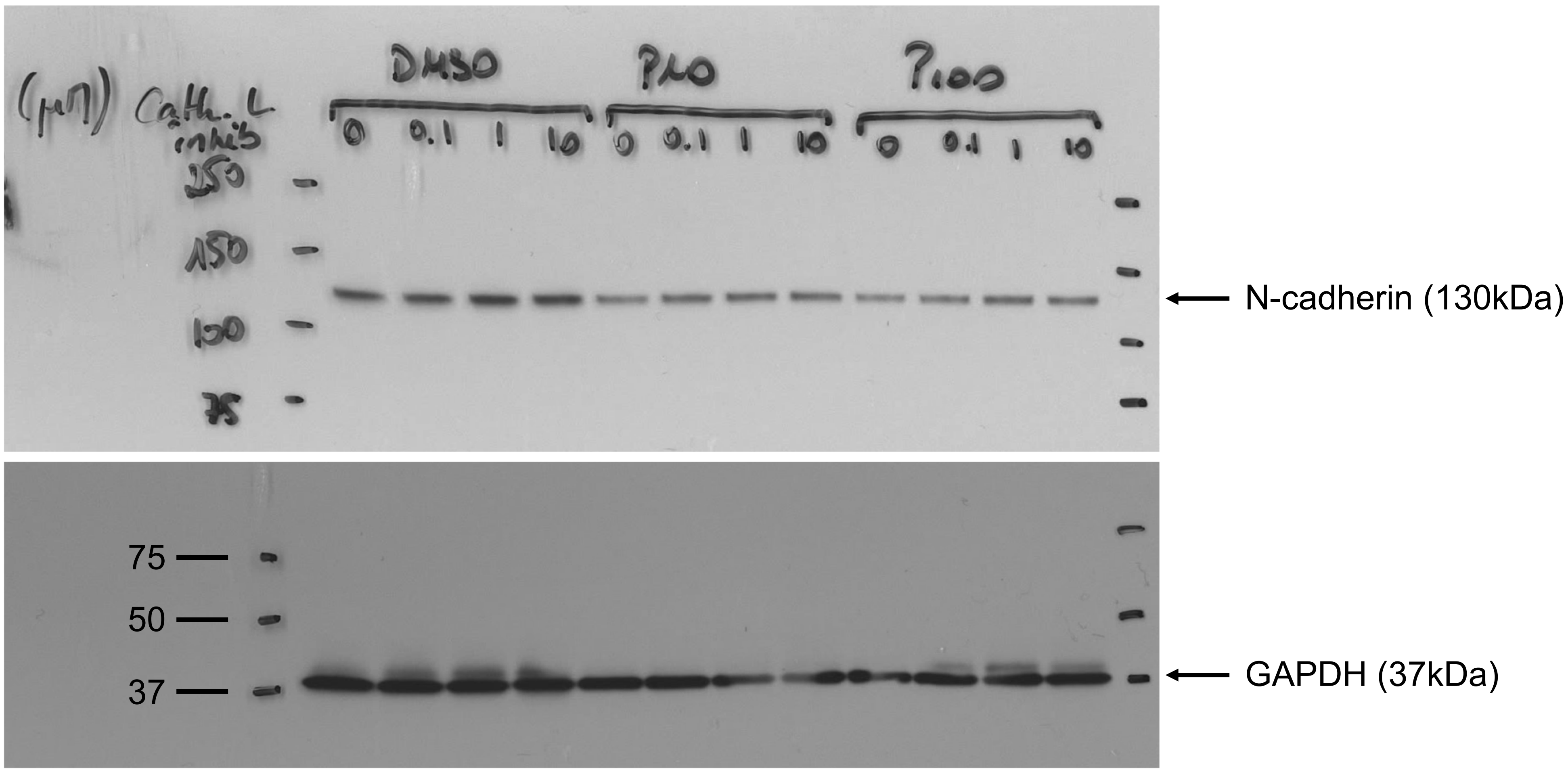
DMSO
(μM) Cath. L inhib
0
0.1
1
10
250
150
100
75
N-cadherin (130kDa)
75
50
37
GAPDH (37kDa)

Figure 8 (3/3)

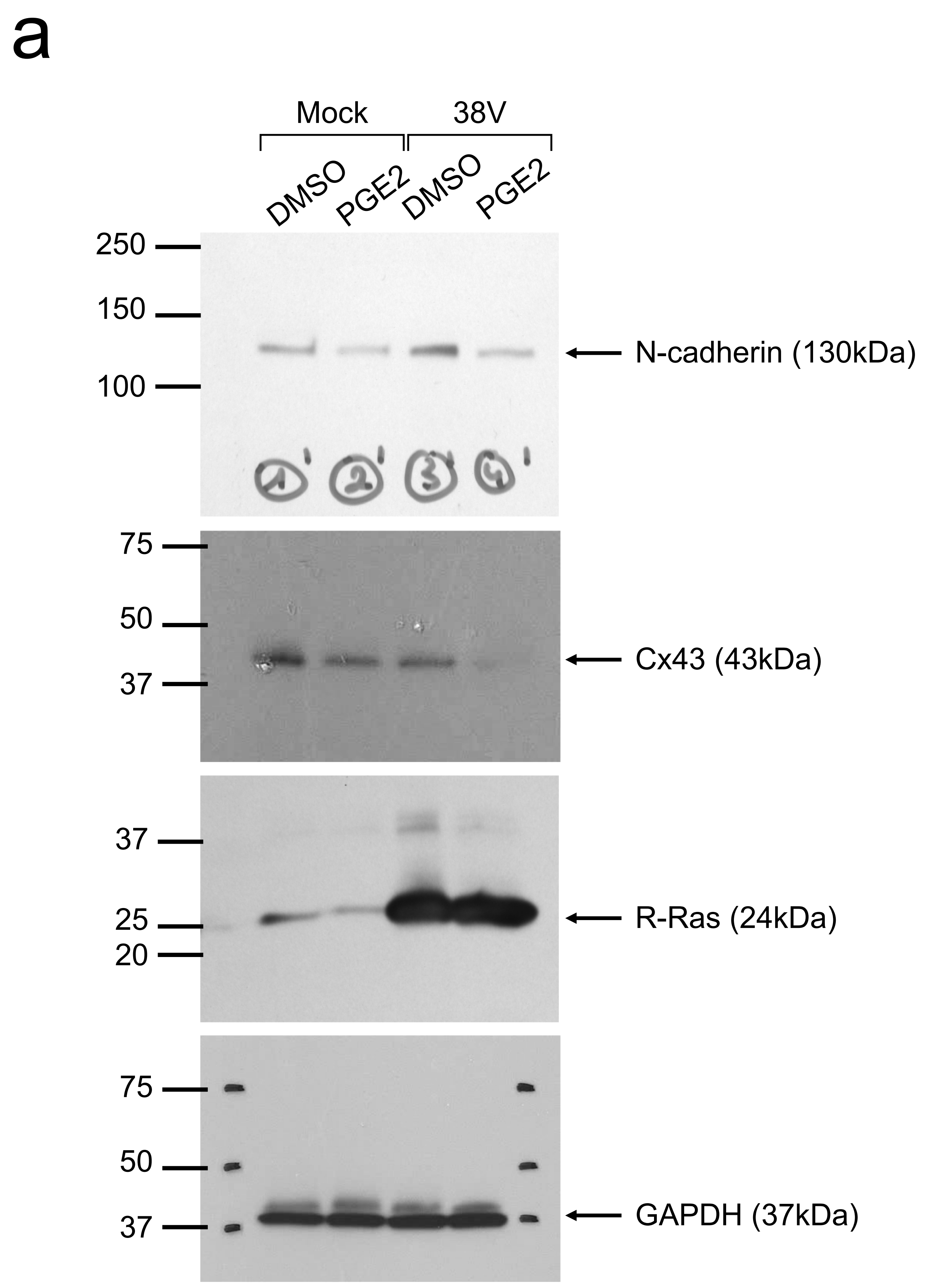
a
Mock
38V
DMSO
PGE2
DMSO
PGE2
250
150
100
N-cadherin (130kDa)
75
50
37
Cx43 (43kDa)
37
25
20
R-Ras (24kDa)
75
50
37
GAPDH (37kDa)

Figure 9

C

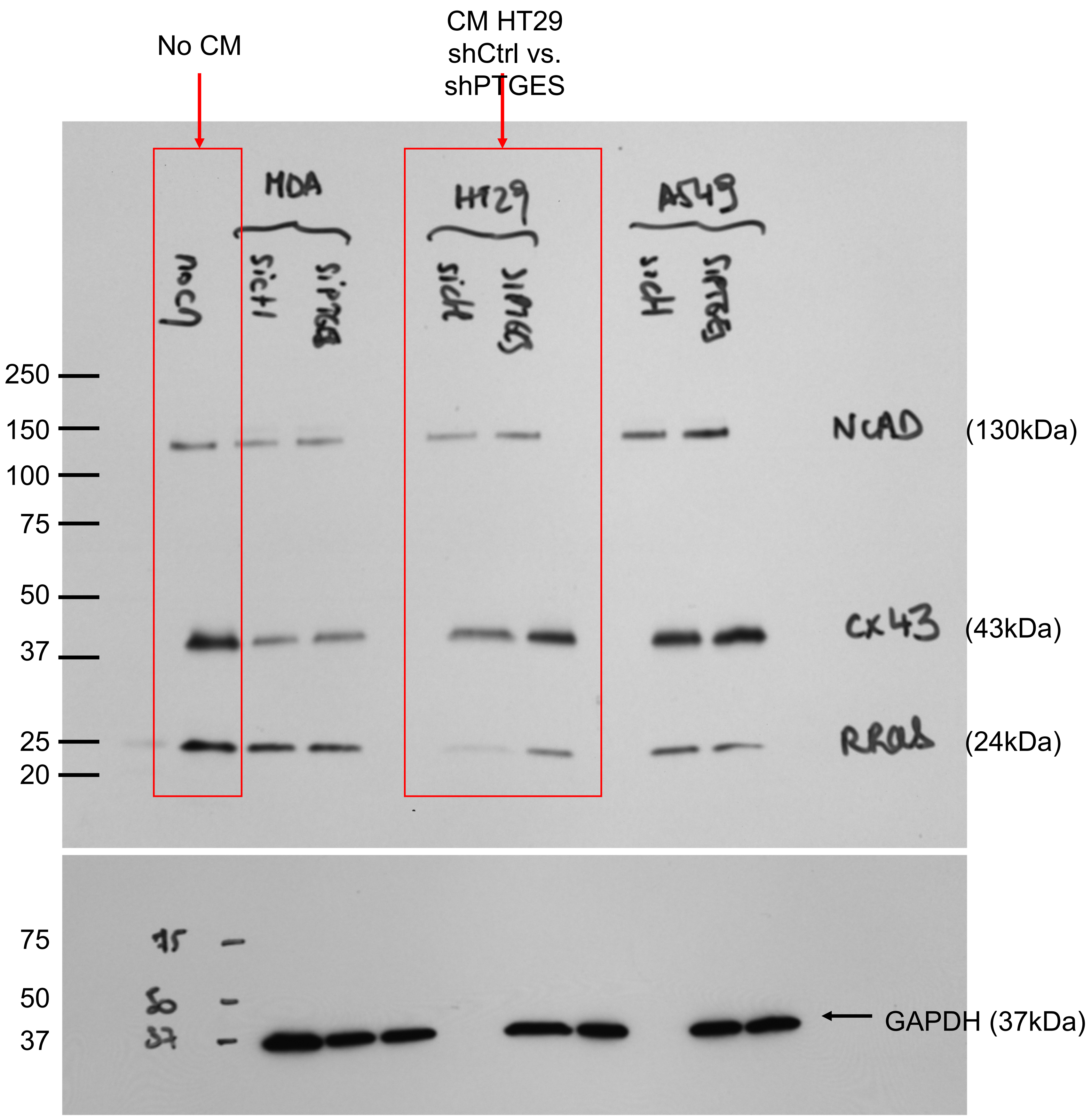
No CM
CM HT29
shCtrl vs.
shPTGES
250
150
100
75
50
37
25
20
NCAD
(130kDa)
Cx43
(43kDa)
RRas
(24kDa)
75
50
37
GAPDH (37kDa)

Figure 10

C

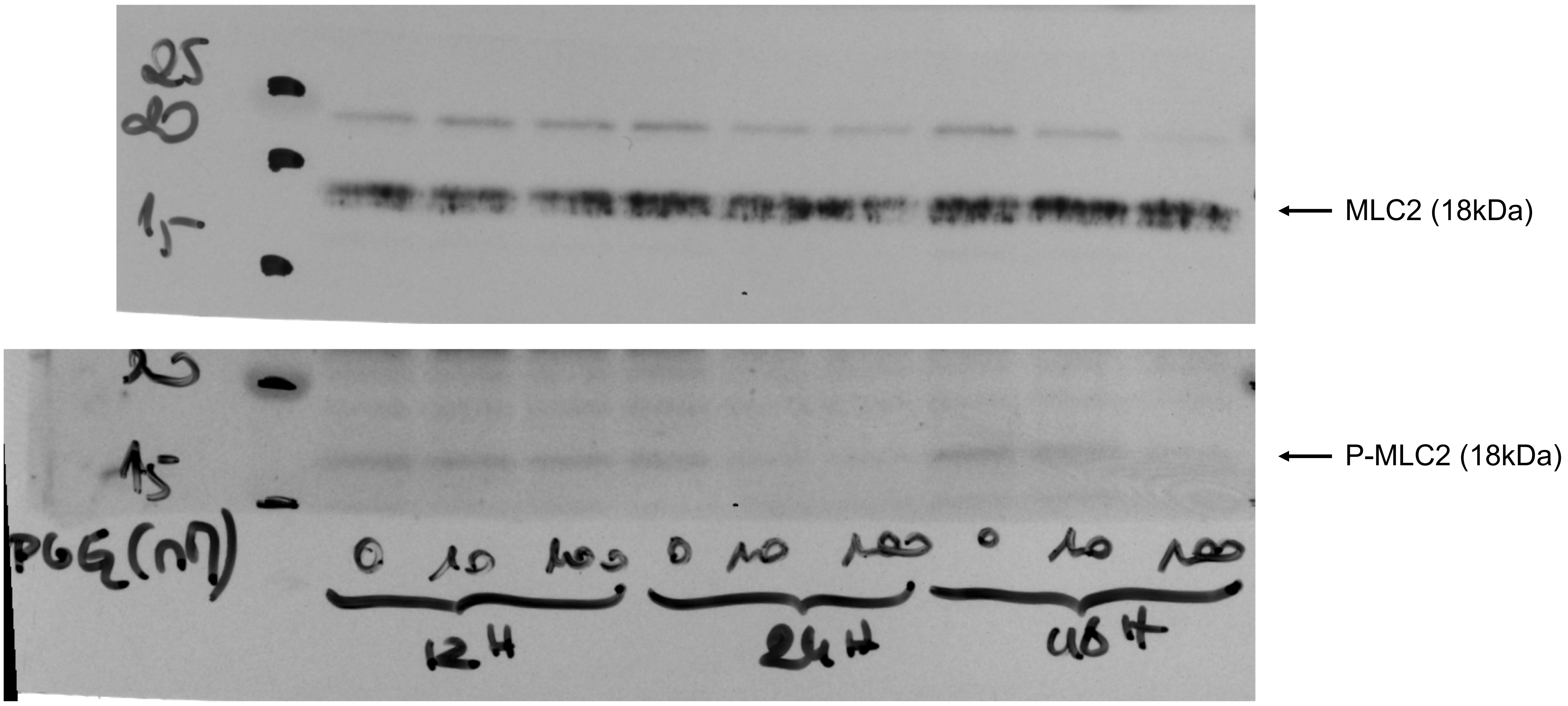

Supplementary Figure 1

b

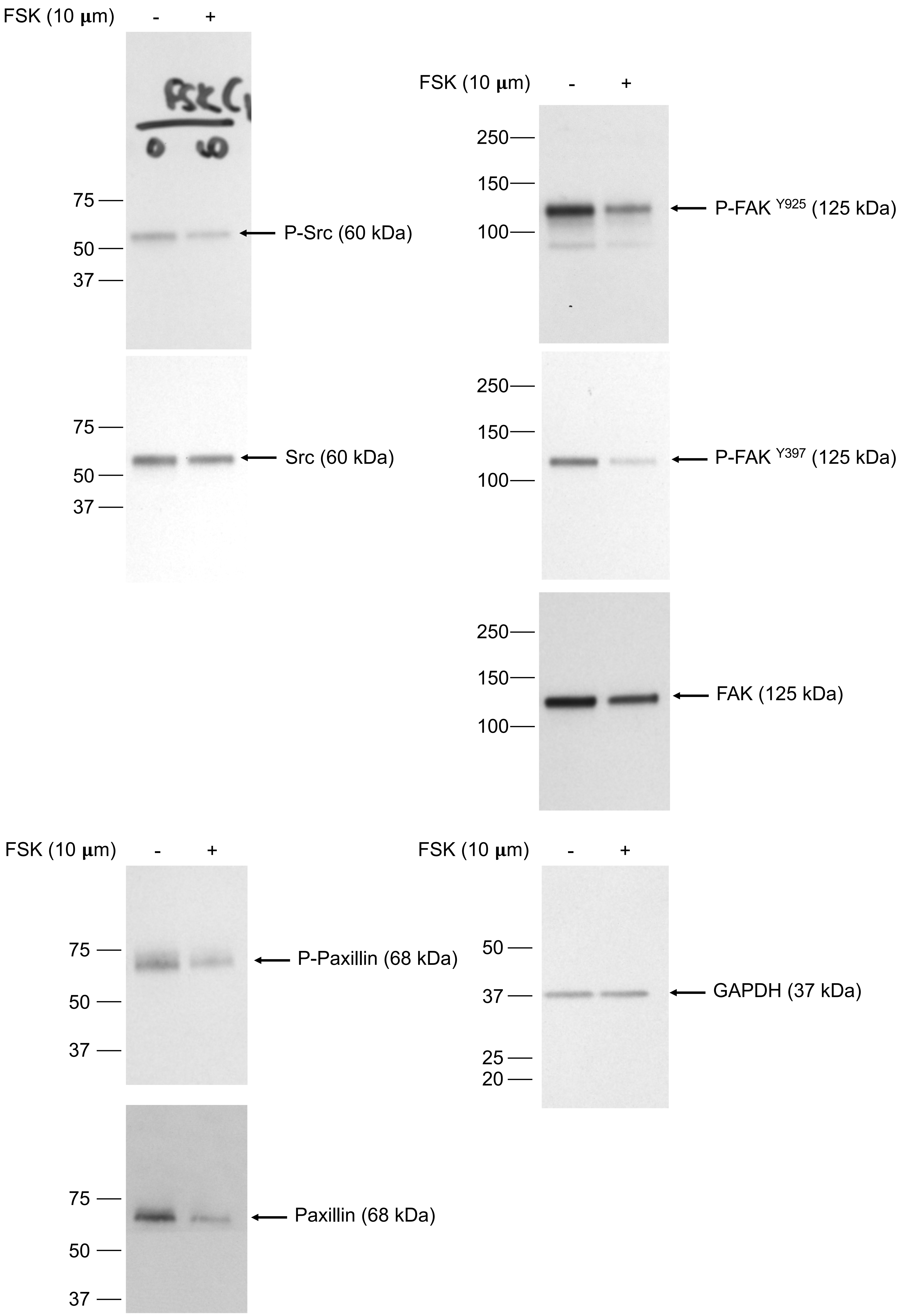

Supplementary Figure 2
